# Proline-Adjacent Phosphosites on *Saccharomyces cerevisiae* Histone Demethylase Rph1p are Salt Stress Responsive and Important for Cell Growth Under Salt Stress

**DOI:** 10.1016/j.mcpro.2025.101066

**Published:** 2025-09-08

**Authors:** Nicola M. Karakatsanis, Joshua J. Hamey, Marc R. Wilkins

**Affiliations:** 1 Systems Biology Initiative, School of Biotechnology & Biomolecular Sciences, UNSW Sydney, Australia; 2 ARC Centre of Excellence for the Mathematical Analysis of Cellular Systems, UNSW Sydney, Australia

**Keywords:** histone methylation, histone demethylase, phosphorylation, yeast salt stress response, snoRNA

## Abstract

Phosphorylation of histone lysine demethylases is an important mechanism by which the cell modulates chromatin dynamics to regulate its response to stress. There is evidence that the *Saccharomyces cerevisiae* H3K36me2/3 demethylase, Rph1p, is an integrator of many signaling events. However, the regulatory function of most Rph1p phosphosites in stress response pathways remains unknown. Here, we investigated the role of Rph1p phosphorylation in the salt stress response. We showed that Rph1p is phosphorylated at seven sites in response to acute high salt stress, most of which are proline-adjacent. Genomic phosphonull mutations identified four salt-stress responsive phosphosites—S410, T411, S412, and S689—to be important for yeast cell growth in this condition. Phosphonull mutations at S412 or S689 were not associated with changes in the proteome in the chronic salt stress response. However, the Rph1p-S689A mutant downregulated a subset of 18 snoRNA genes in chronic salt stress compared to the wildtype, an effect absent in the Rph1p-S412A mutant. The downregulation of several snoRNA may cause changes to ribosomal RNA modifications and affect ribosome function. Consistent with these targeted transcriptional changes, neither mutant was associated with gross changes in H3K36 methylation in chronic salt stress. These findings suggest that S689 phosphorylation directs Rph1p to specific regions of the chromatin in the chronic salt stress response. Overall, our findings identify S689 as a key phosphorylation site linking Rph1p to salt stress-responsive gene regulation, offering new insights into stress-responsive mechanisms in eukaryotes.

## Highlights

• Histone demethylase Rph1p shows extensive salt stress-responsive phosphorylation.
• Phosphonull mutations of Rph1p impaired growth in salt stress.
• Rph1p phosphonull mutations did not affect the proteome in chronic salt stress.
• Phosphonull mutations of Rph1p did not affect the gross methylation of H3K36.
• Rph1p phosphonull S689A affected snoRNA gene transcription in chronic salt stress.

## Graphical Abstract

**Figure:**
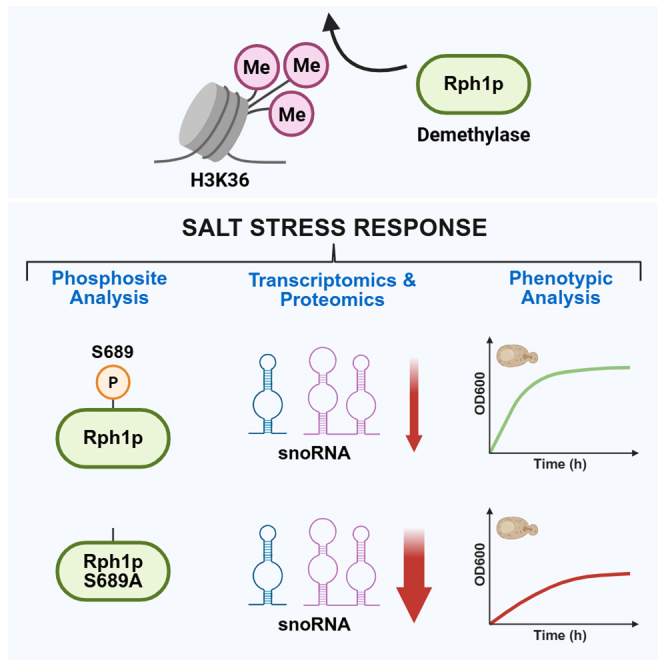

## In Brief

Phosphorylation of histone demethylases is an important mechanism for modulating chromatin dynamics in response to stress. Yeast H3K36me2/3 demethylase, Rph1p, is extensively phosphorylated in stress however the function of most phosphosites remains unknown. Mutation of phosphosites showed their importance for cell growth in salt stress. Investigation of the proteome and transcriptome of mutants revealed the S689 phosphosite to be critical for snoRNA gene regulation. Our findings highlight that phosphorylation sites in histone demethylating enzymes can regulate expression of specific genes.

**Fig. 1:**
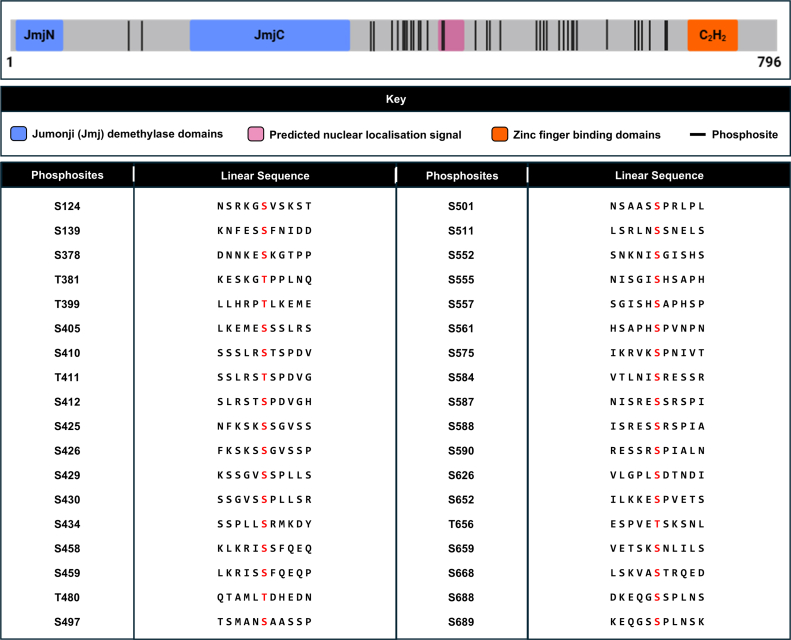
**Rph1p structural features and phosphosites.** Rph1p has a JmjN demethylase domain (aa 14–55) (*blue*), JmjC demethylase domain (aa 193–355) (*blue*), putative bipartite nuclear localisation signal (aa 455–471) (*pink*) and two C_2_H_2_-type zinc finger domains (aa 709–732; 738–763) (*orange*) [UniProt: P39956]. Outside of these characterised domains/regions, Rph1p has low predicted secondary and tertiary structure, with extensive regions of predicted intrinsic disorder at the C-terminus. Rph1p has 36 phosphorylated amino acids (*black lines*), all located outside its folded domains, primarily in predicted intrinsically disordered regions. The linear sequence (pS/T ± 5 aa) of these sites are tabulated, with phosphosites given in *red*.

In *Saccharomyces cerevisiae*, histone lysine methylation is regulated by four methyltransferases (Dot1p, Set1p, Set2p and Set5p) and four demethylases (Gis1p, Jhd1p, Jhd2p and Rph1p) (1). Rph1p catalyzes the removal of di- and tri-methyl on lysine 36 of histone H3 (H3K36) and is a human KDM4-family homolog (2, 3, 4, 5, 6). Unlike KDM4-family demethylases, Rph1p has no other known substrates besides H3K36. In terms of domain architecture (Fig. 1), Rph1p has a Jumonji (Jmj) N domain, and a JmjC domain which contains the catalytic H x H···H triad and part of the substrate binding cleft (5). The JmjN domain associates with the JmjC domain and is required for the catalytic activity of Rph1p. Rph1p also has two C-terminal C_2_H_2_ zinc finger (ZF) domains which are not required for catalytic activity *in vitro*, but may target Rph1p to specific regions of the genome *in vivo* (4). Unlike many other chromatin-remodeling/modifying enzymes, Rph1p is not known to be part of a heterologous high molecular weight protein complex (4). However, size exclusion analysis suggests that it forms a homo-oligomer comprised of four Rph1p subunits (4). Efforts to discover Rph1p structural conformations are complicated by its highly intrinsically disordered regions outside of the characterized catalytic core and ZF domains and the lack of known stable interaction partners (Fig. 1).

Rph1p functions primarily as a transcriptional repressor during optimal conditions, with a large proportion of Rph1p-repressed genes belonging to the DNA damage response or involved in the characterized “environmental stress response” (7, 8, 9, 10, 11, 12, 13). The RPH1 gene is non-essential and when knocked out, cells are morphologically wildtype and gross H3K36 methylation levels are unaffected (4). However, a significant increase in H3K36me3 at several genes (*e.g.* FMP27, PMA1, ADH1, and YEF3) has been observed (3, 6), suggesting that Rph1p demethylase activity is or can be targeted. The over-expression of RPH1 causes growth defects which are dependent on its ZF domains (4), but how this affects Rph1p localization to chromatin or gross methylation at H3K36 remains unknown.

Phosphorylation of histone lysine demethylases (KDMs) is an important mechanism by which the cell modulates chromatin dynamics to regulate its response to stress. In particular, phosphorylation is known to influence histone KDM function by changing protein subcellular localisation or chromatin binding, altering protein half-life or changing protein complex formation or dissociation (14). This can alter histone methylation, which can influence gene expression patterns and ultimately affect cellular behavior and phenotype. An example of this is the phosphorylation of human KDM3A at S265 in subcutaneous white adipose tissue in response to chronic cold stress. Phosphorylation triggers formation of the KDM3A–PPARγ–PGC1α–PRDM16 complex, whereby KDM3A is recruited to the enhancers/promoters of thermogenic beige-selective genes, and demethylates H3K9me2 to facilitate gene induction (15).

We have previously shown *S. cerevisiae* Rph1p to have at least 36 phosphorylated amino acids (Fig. 1) (1). Some of these phosphorylation sites have been functionally investigated by others. For instance, the Rph1p-S412A phosphonull mutant shows substantially reduced binding to chromatin in response to SAM depletion (16). Rph1p-S412 phosphorylation is dispensable for enzymatic activity but likely facilitates net demethylation of H3K36me by mediating Rph1p association with chromatin (16). In contrast, Rph1p-S652A has impaired dissociation from the promoter of PHR1 upon UV irradiation. It is proposed that dissociation of phosphorylated Rph1p releases the Rpd3p co-repressor complex facilitating PHR1 transcription (7, 17). Separately, it has been shown that phosphonull co-mutants of several Rph1p sites (T411, S412, S425, S426, S429, S430, S557 and S561) partially inhibits phosphorylation of Rph1p and autophagy induction upon nitrogen starvation. Thus, it is suggested that phosphorylation at one or more of these sites inhibits Rph1p repression of autophagy genes, which is prerequisite for autophagy induction (18). Correspondingly, Rph1p phosphomimetic mutants of S412, S425/S426, S429/S430, S434, and S557 exhibited greater resistance to rapamycin, which artificially induces a starvation-like state (13). A phosphomimetic of all the aforementioned sites combined exhibited greater resistance to rapamycin and decreased association with ribosome-related genes, both in the presence and in the absence of rapamycin (13). Consequently, phosphorylation of Rph1p at these sites likely mediates its dissociation from the chromatin at ribosome-related genes, thereby promoting cell survival upon nutrient stress conditions. From the above, there is strong evidence that Rph1p is an integrator of many signaling events. However, we have a limited understanding of the regulatory function of most Rph1p phosphorylation sites in stress response pathways.

To identify phosphosites involved in stress responses, we quantified Rph1p phosphorylation in yeast cells subject to salt and caffeine stress. Rph1p became extensively phosphorylated at seven sites in response to salt stress but was less responsive to caffeine. Mutagenesis of salt stress-responsive phosphosites identified those important for cell growth in salt stress. Two phosphonull mutant strains, Rph1p-S412A and Rph1p-S689A, were further investigated using quantitative mass spectrometry and RNA-sequencing to assess changes in protein abundance and gene expression, respectively. Loss of S689 phosphorylation specifically reduced expression of a subset of snoRNA genes in chronic salt stress, whilst the Rph1p-S412A mutation had little effect on the proteome or transcriptome. Targeted mass spectrometry showed that gross H3K36 methylation was not strongly affected by the absence of phosphorylation in chronic salt stress, reflecting the targeted changes seen in the transcriptome. These findings show that S689 phosphorylation is an important regulator of Rph1p function in the yeast salt stress response.

## Experimental Procedures

Primers and plasmids used throughout this study are outlined in Supplemental Tables S1 and S2, respectively.

### Rph1p and Set2p Phosphorylation Assays Under Stress Conditions

*S. cerevisiae* BY4741 cells containing the galactose-inducible expression vector pD1204 with either C-terminally 6× histidine tagged RPH1 or SET2 (Supplemental Table S2) were grown in synthetic complete (SC) broth without uracil (6.7 g/L yeast nitrogen base with ammonium sulphate without amino acids, 1.92 g/L synthetic drop out mix without uracil and 20 g/L D-glucose) at 30 °C until 0.8 OD_600_. Expression of RPH1 and SET2 was induced in YP broth (10 g/L yeast extract and 20 g/L peptone) supplemented with 20 g/L D-galactose at 30 °C for 9 h. In the last 15 min of expression, cells were incubated with 1 M NaCl or 15 mM caffeine for pD1204-RPH1-containing cells or 1 M NaCl for pD1204-SET2-containing cells. Cells were resuspended in binding buffer (46.6 mM Na_2_HPO_4_, 3.4 mM NaH_2_PO_4_, 500 mM NaCl, 40 mM imidazole and 0.25% *(v/v)* Triton-X-100, pH 8) supplemented with cOmplete EDTA-free protease inhibitor cocktail (Roche) and PhosSTOP pan-phosphatase inhibitor (Roche) and lysed using a Mini-Beadbeater-24 (BioSpec Products) for three cycles of 1 min shaking and incubation on ice for 3 min between. Lysates were clarified by centrifugation at 4 °C for 40 min at 21000*g*. Rph1p and Set2p were purified by their 6× histidine tag using a HisTrap FF column prepacked with Ni^2+^ sepharose (Cytiva). Columns were washed with 10 ml wash buffer (46.6 mM Na_2_HPO_4_, 3.4 mM NaH_2_PO_4_, 500 mM NaCl and 40 mM imidazole, pH 8) (1 ml/min) before proteins were eluted with 6 ml elution buffer (46.6 mM Na_2_HPO_4_, 3.4 mM NaH_2_PO_4_, 500 mM NaCl and 500 mM imidazole, pH 7.4) (1 ml/min). Eluted proteins were concentrated using Amicon Ultra-4 10K centrifugal filters (Merck Millipore).

Purification fractions were run on an SDS-PAGE and Rph1p and Set2p gel bands were excised for mass spectrometry analysis. Excised gel bands were destained with 50% *(v/v)* acetonitrile/50% *(v/v)* 20 mM NH_4_HCO_3_ and dehydrated with 100% acetonitrile. Proteins were subsequently reduced with 5 mM dithiothreitol (DTT) for 30 min at 37 °C and alkylated with 10 mM iodoacetamide (IAA) for 30 min in the dark at room temperature. In-gel proteolytic digestion of proteins was conducted with 50 ng Trypsin (sequencing grade, Promega) in 20 mM NH_4_HCO_3_ overnight at 37 °C. Peptides were extracted from gel bands with 50% *(v/v)* acetonitrile/50% 0.1% (*v/v*) formic acid for 30 min followed by 100% acetonitrile for 10 min. Peptides were vacuum dried and resuspended in 0.1% (*v/v*) formic acid for injection onto the mass spectrometer.

Mass spectra were acquired on the Q Exactive Plus (Thermo Scientific) with data-dependent acquisition (DDA) using a 32 min gradient from 2% buffer B at 4 min to 45% buffer B at 36 min. Precursor scans (range: 350–1750 *m/z*) were acquired in the Orbitrap (resolution = 70,000) with an automated gain control (AGC) target of 1 × 10^6^ ions and maximum injection time of 50 ms. The 10 most abundant precursor ions were isolated in the quadrupole (isolation window = 2.5 *m/z*) and fragmented by higher-energy collisional dissociation (HCD) (normalized collision energy (NCE) = 30 eV). Resulting fragment ions were analyzed in the Orbitrap (resolution = 17,500) with an AGC target of 1 × 10^5^ and maximum injection time of 125 ms. Dynamic exclusion was enabled with an exclusion duration of 30 s.

Raw files (.raw) were converted to Mascot Generic Files (.mgf) for database searching using RawConverter (version 1.2.0.1). Mass spectra were searched against the SwissProt 2023_04 database (570,157 sequences) (Rph1p) or SwissProt 2023_05 database (570,420 sequences) (Set2p) using the Mascot MS/MS Ions Search (version 2.8) with the following parameters: taxonomy (*S. cerevisiae*), missed cleavages (2), enzyme (trypsin), variable modifications (carbamindomethyl (C), oxidation (M), phospho (S/T), phospho (Y)), peptide tolerance (4 ppm), MS/MS tolerance (20 ppm), and peptide charge (2+, 3+, 4+). Mascot-generated DAT files were imported into Skyline (version 24.1.0.199) to generate extracted ion chromatograms (XICs). XIC peak boundaries were manually curated based on retention time and MS2 profile and label-free quantification performed using the area under the curve. The relative phosphorylation was determined by calculating the amount of the phosphorylated peptide compared to the total amount of the unphosphorylated and phosphorylated peptide. In cases where two separate sites on the same peptide were phosphorylated and the respective peptides eluted as distinct peaks, relative phosphorylation was determined by calculating the amount of the phosphorylated peptide compared to the total amount of the unphosphorylated peptide and all other singly phosphorylated versions of the peptide. Some peptides were represented by more than one site because the phosphorylation event could not be localized by MS2 or there was evidence that it was a combination of sites where the respective phosphopeptides co-eluted during LC-MS/MS. Some sites were represented more than once due to missed cleavages resulting in alternative forms of the phosphopeptide which were separately quantified. Quantified Rph1p and Set2p peptides are shown in Supplemental Tables S3 and S4, respectively. MS2 spectra for localization of phosphosites in Rph1p and Set2p quantified phophopeptides are shown in Supplemental Figs. S1 and S2, respectively.

### *In Vivo* Gross H3K36 Methylation Assays

For yeast cells subject to acute salt stress, 12 ml cultures were grown at 30 °C to 0.8 OD_600_ in SC broth (6.7 g/L yeast nitrogen based with ammonium sulfate without amino acids, 1.92 g/L synthetic drop out mix without uracil, 80 mg/L uracil and 20 g/L D-glucose) before being incubated with or without 1 M NaCl for 15 min. For chronic salt stress, 12 ml yeast cultures were growth in SC broth with or without 1 M NaCl for ∼2 to 3 doublings at 30 °C until 0.8 OD_600_. Cells were lysed as above in buffer containing 50 mM 4-(2-Hydroxyethyl)piperazine-1-ethane-sulfonic acid (HEPES), 2 mM DTT, 100 mM NaCl, 2 mM EDTA, 0.5% Triton-x-100 (pH 7) supplemented with cOmplete EDTA-free protease inhibitor cocktail (Roche). Lysates were clarified by centrifugation at 4 °C for 40 min at 4000*g*. Clarified lysates were run on an SDS-PAGE and gel bands corresponding to the size of the histone H3 protein were excised. To ensure consistent peptides containing K36 were obtained, histones were subject to in-gel derivatization by propionic anhydride as described previously in (1). In-gel proteolytic digestion of proteins was conducted with 50 ng Trypsin (sequencing grade, Promega) in 20 mM NH_4_HCO_3_ overnight at 37 °C and peptides were extracted as above.

Mass spectra were acquired on the Q Exactive Plus (Thermo Scientific) with DDA followed by parallel reaction monitoring (PRM) using a 32 min gradient from 2% buffer B at 4 min to 45% buffer B at 36 min. Precursor scans (range: 400–900 *m/z*) were acquired in the Orbitrap (resolution = 70,000) with an AGC target of 3 × 10^6^ and maximum injection time of 100 ms. For PRM, the inclusion list contained the *m/z* of both doubly and triply charged differentially methylated versions of the H3K36 peptide (KSAPSTGGVKKPHR), accounting for the additional mass shift due to propionylation of un- and/or mono-methylated lysine (Table. 1). Precursor ions were isolated in the quadrupole (isolation window 1.4 *m/z*) and fragmented by HCD (NCE = 30 eV). Resulting fragment ions were analyzed in the Orbitrap (resolution = 17,500) with an AGC target of 2 × 10^5^ and maximum injection time of 100 ms. Dynamic exclusion was enabled with an exclusion duration of 30 s. This analysis complies with Tier 3 Molecular and Cellular Proteomics guidelines for targeted MS analysis.

**Table 1:** Inclusion list used for parallel reaction monitoring of H3K36 peptides

Peptide^a^	State	2+ *m/z*	3+ *m/z*
K(Pr/56.03)SAPSTGGVK(Pr/56.03)K(Pr/56.03)PHR	me0	809.45720	539.97389
K(Pr/56.03)SAPSTGGVK(Me/14.012)K(Pr/56.03)PHR	me1	788.45191	525.97037
K(Pr/56.03)SAPSTGGVK(Pr/56.03 + Me/14.012)K(Pr/56.03)PHR	me1	816.46502	544.64577
K(Pr/56.03)SAPSTGGVK(Di/28.03)K(Pr/56.03)PHR	me2	795.45974	530.64225
K(Pr/56.03)SAPSTGGVK(Tri/42.05)K(Pr/56.03)PHR	me3	802.46756	535.31413

Di, dimethyl; Me, monomethyl; Pr, propionyl; Tri, trimethyl.

a Modification/mass shift is listed in ().

Raw files (.raw) were imported into Skyline (version 24.1.0.199) and XIC peak boundaries for PRM peptides were manually curated based on retention time and MS2 profile. Label-free quantification was performed using the area under the curve of triply charged peptides. The relative methylation state was determined by calculating the amount of the methylated peptide compared to the total amount of the unmethylated peptide and all other differentially methylated versions of the peptide. Statistical analysis was performed using an ordinal logistic model with proportional odds assumption (Supplemental Figs. S3 and S4).

### Liquid Growth Assays of Mutant Strains

Liquid growth assays were performed on *S. cerevisiae* strains of interest using a 96-well plate configuration. Cells were grown in SC broth at 30 °C for three doublings until ∼0.4 OD_600_. A 96-well plate containing SC broth supplemented with different concentrations of NaCl was inoculated with yeast cells at ∼0.1 OD_600_. Final concentrations of NaCl used were 0 M, 0.5 M, 0.8 M and 1 M as indicated. Plates were incubated at 30 °C in a CLARIOstar microplate reader (BMG Labtech) with shaking until saturation and OD_600_ nm was measured every 10 minutes. Doubling times were calculated using the steepest slope of the Log_10_ transformed growth curve.

### Site Directed Mutagenesis of RPH1

Site directed mutagenesis of RPH1 phosphosites was conducted on the pRS426 shuttling vector using the primers outlined in Supplemental Table S1, as per (1, 19). Mutant plasmids were verified by Sanger sequencing using the relevant sequencing primers in Supplemental Table S1. Mutated RPH1 sequences together with the URA3 auxotrophic selection marker were amplified from pRS426 plasmids with overhangs homologous to regions immediately upstream and downstream of the genomic RPH1 coding sequence. PCR products were subsequently transformed into the wildtype *S. cerevisiae* strain BY4741, as outlined in (20), for homologous recombination into the genome. Incorporation of desired mutations at the RPH1 loci were confirmed by Sanger sequencing of amplicons derived from genomic DNA (see Supplemental Table S1 for primers).

### 2nSILAC Proteome Profiling of Rph1p Phosphonull Mutants

Stable isotope labeling by amino acids in cell culture (SILAC) was used for proteome profiling of *S. cerevisiae* strains of interest. The 2nSILAC method (21, 22) was used whereby cells were metabolically labelled with either heavy isotopes of arginine (^13^C_6_^15^N_4_/+ 10 Da) and lysine (^13^C_6_^15^N_2_/+ 8 Da) or light isotopes of arginine and lysine. Yeast seed cultures were grown for ∼12 doublings at 30 °C until 0.8 OD_600_ in minimal media (6.7 g/L yeast nitrogen base with ammonium sulfate without amino acids, 20 g/L D-glucose, 20 mg/L L-histidine, 20 mg/L L-leucine, 20 mg/L L-methionine, 20 mg/L L-tryptophan, 20 mg/ml D-adenine, 20 mg/L D-uracil, 200 mg/L L-proline). For heavy-labelled cells, media was supplemented with 287 μM ^13^C_6_^15^N_4_ (Arg10) L-arginine monohydrochloride and 342 μM ^13^C_6_^15^N_2_ (Lys8) L-lysine monohydrochloride (Silantes). Excess L-proline was used to minimize the metabolic conversion of heavy L-arginine to heavy L-proline (^13^C_5_^13^N_1_, Pro6). Equivalent molarity L-arginine monohydrochloride and L-lysine monohydrochloride was used for light labelled cells. From here, 30 ml cultures containing minimal media with either heavy- or light-labelled arginine and lysine, supplemented with 1 M NaCl, were grown for ∼2 to 3 doublings at 30 °C until 0. 8 OD_600_.

Cells were lysed in denaturing lysis buffer (100 mM NH_4_HCO_3_, 8 M urea, pH 8) by glass bead homogenization as above and pairwise comparisons of wildtype and mutant strains were conducted by mixing differentially labelled lysates 1:1 (*w/w*) to 400 μg total protein. Label swap experiments were conducted giving two pairwise comparisons per mutant strain. Proteins were reduced with 5 mM DTT for 30 min at 37 °C and alkylated with 10 mM IAA for 30 min in the dark at room temperature, before being diluted tenfold in 50 mM NH_4_HCO_3_. Trypsin (sequencing-grade, Promega) was used for overnight proteolytic digestion at 1:100 proteinase: protein (*w/w*) ratio at 37 °C. Peptides were desalted using Sep-Pak tC18 cartridges (Waters) as described in (23). The mean heavy-labelled proline conversion rate was 6.3% and the mean heavy-labelled incorporation of arginine and lysine was >99.2% for all strains (Supplemental Table S5).

Peptides were fractionated using the Agilent 1260 Infinity II HPLC system or Agilent 1290 Infinity ultra-performance liquid chromatography system coupled to a Phenomenex Luna five μm SCX 100 Å (250 x 100 mm) liquid chromatography column with buffer A (7 mM KH_2_PO_4_ (pH 2.6), 30% (*v/v*) acetonitrile) and buffer B (7 mM KH_2_PO_4_ (pH 2.6), 30% (*v/v*) acetonitrile, 350 mM KCl). The following gradient was used at a flow rate of 1.5 ml/min with fractions collected every minute: 0% B at 0 min, 5% B at 7.5 min, 60% B at 47.5 min, 100% B at 67.5 min, 100% B at 77.5 min, 0% B at 77.51 min. For mass spectrometry analysis, individual 1 min fractions were pooled into five larger fractions from the following elution times: F1 = 20 to 41 min, F2 = 41 to 51/52 min, F3 = 51/52-63 to 64 min, F4 = 63/64 to 75/76 min and F5 = 75/76 to 88 min. Pooled fractions were desalted using Sep-Pak tC18 cartridges (Waters) as previously described (23) before being vacuum dried and resuspended in 0.1% (*v/v*) formic acid for injection onto the mass spectrometer.

Mass spectra of fractionated peptides were acquired on the Fusion Lumos Tribrid (Thermo Scientific) with DDA using a 125 min gradient from 2% buffer B at 4 min to 45% buffer B at 36 min. Precursor scans (range: 350–1750 *m/z*) were acquired in the Orbitrap (resolution = 120,000). Precursor ions were isolated in the Quadrupole with a 1.6 *m/z* isolation window and fragmented by HCD (NCE = 30 eV). The resulting fragment ions were analyzed in the Linear Ion Trap with a rapid scan rate. The cycle time was set to 3 s, and dynamic exclusion was enabled with an exclusion duration of 25 s.

Mass spectra were searched against the *S. cerevisiae* database (UP000002311) with decoys and common contaminants using the Fragpipe (22.0) SILAC3 workflow. MSFragger was run with strict trypsin for protein digestion (missed cleavages = 2), precursor tolerance of 20 ppm and fragment ion tolerance of 20 ppm with recalibration. Modifications of carbamidomethyl (C) (fixed), oxidation (M) (variable), acetylation (N-term) (variable), Arg10 (R) (variable), Lys8 (K) (variable) and Pro6 (P) (variable) were selected. IonQuant was used for MS1 quantification with heavy labeling Arg10 and Lys8, and light labeling Arg0 and Lys0. Match between runs was enabled using a 1% ion false discovery rate (FDR). The combined protein median Log2 heavy-to-light (H/L) ratios were normalized and the reverse label multiplied by −1 to obtain all fold change protein abundance ratios as mutant: wildtype. Proteins identified by less than <2 peptides and contaminant/decoy proteins were removed. Proteins for which ratios were obtained in both biological replicates were analyzed in Perseus (2.0.11). The distribution of the filtered normalized median Log2 H/L ratios for each biological replicate of the Rph1p-S412A and Rph1p-S689A experiments are shown in Supplemental Fig. S5. The filtered normalized median Log2 H/L ratios for each biological replicate of the Rph1p-S412A and Rph1p-S689A experiments are in Supplemental Tables S6 and S7. Statistical analysis was performed on these proteins using a one sample *t-*test (use for truncation: Benjamini-Hochberg FDR, *q*-value < 0.05) where the mean normalized median Log2 fold change in protein abundance in the mutant strain relative to the wildtype strain was compared to the hypothetical value of 0 (no change).

### RNA-Sequencing of Rph1p Wildtype and Mutant Strains

For wildtype and RPH1 mutant cells subject to acute salt stress, 5 ml cultures were grown at 30 °C to 0.8 O D_600_ in SC broth before being incubated with 1 M NaCl for 15 min. For chronic salt stress, 5 ml yeast cultures were growth in SC broth with 1 M NaCl for ∼2 to 3 doublings at 30 °C until 0.8 OD_600_. All cultures and downstream analyses were performed in biological triplicate. Total RNA extraction was performed using the Monarch Total RNA Miniprep Kit (New England Biolabs). Library preparation was performed using the Illumina Stranded mRNA prep and sequencing conducted on the NovaSeq X Plus (Illumina) with 10 B reads and 2 x 100 bp read length. Quality control was performed on all raw forward reads using FastQC (version 0.12.1) and Trim Galore! (version 0.6.10) was used to remove low-quality reads and adaptor contamination. An average of 29.5 million filtered reads per sample were mapped to the *S. cerevisiae* R64-1-1 genome (GCA_000146045.2) using STAR (version 2.7.10 b) and quantified with Subread (version 2.0.8). Genes where the summed read count across samples was ≤ 1 were removed and DESeq2 (version 1.46.0) was used for count normalization and differential gene expression analysis. Gene Ontology (GO) enrichment analysis was performed using Enricher from clusterProfiler (version 4.14.6), with GO term annotations obtained from the Saccharomyces Genome Database (24). For GO enrichment analysis, statistical analysis was performed using a hypergeometric test with Benjamini-Hochberg FDR correction (adjusted *p*-value <0.05).

### Experimental Design and Statistical Rationale

The number of biological and technical replicates for each experiment is described in the Results. Statistical tests for all analyses are described in the Results.

## Results

### Many Rph1p Phosphosites are Salt Stress-Responsive

**Fig. 2:**
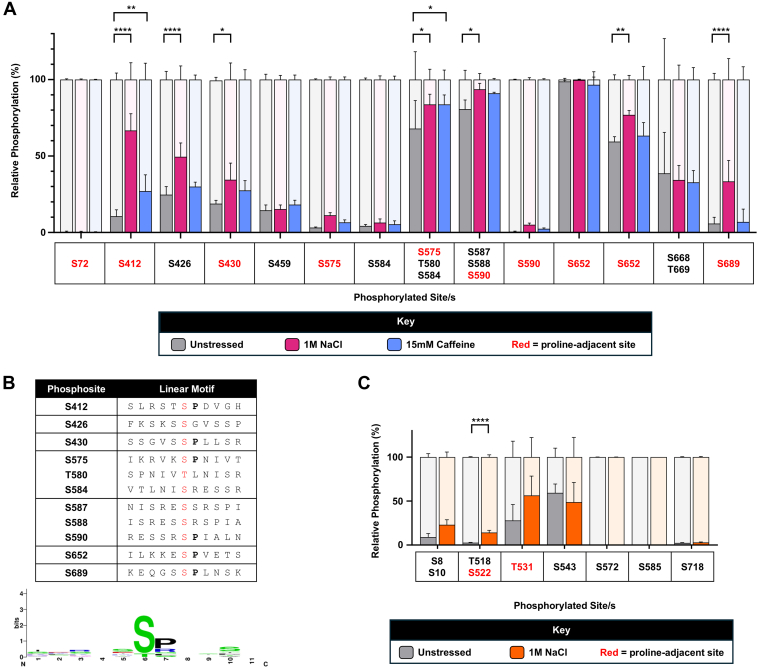
**Many phosphorylation sites on Rph1p are salt stress-responsive**. *A*, change in relative phosphorylation of sites on Rph1p in response to 15 min of 1 M NaCl stress (*pink*) or 15 mM caffeine stress (*blue*). Relative phosphorylation was determined by using LC-MS/MS to measure the amount of the phosphopeptide compared to the total amount of the phosphopeptide and its unphosphorylated counterpart (see Experimental Procedures). Each phosphopeptide is represented by the localized phosphosite/s, as determined manually by the MS2 spectra (Supplemental Fig. S1). Some sites are represented by more than one peptide (missed cleavages), and some peptides represent more than one site (co-elution or ambiguous localization) (see Experimental Procedures). Mean coverage of Rph1p by proteolytic cleavage with trypsin was 57.8%. The mean relative phosphorylation of four biological replicates is shown, and one technical replicate was performed. Statistical analysis was performed using a 2-way ANOVA with multiple comparisons (Dunnet statistical hypothesis testing, adjusted *p*-value < 0.05) to compare the relative abundance of Rph1p phosphopeptides in the 1 M NaCl and 15 mM caffeine treated cells to those in the unstressed cells. *B*, linear sequences and motif of Rph1p phosphosites which significantly increased upon 15 min 1 M NaCl stress. Frequency of amino acids at each position is shown with a WebLogo (81, 82). *C*, change in relative phosphorylation of sites on the H3K36 methyltransferase Set2p in response to 15 min 1 M NaCl stress (*orange*). Relative phosphorylation was determined as for sites on Rph1p. Each phosphopeptide is represented by the localised phosphosite/s, as determined manually by the MS2 spectra (Supplemental Fig. S2). Some sites are represented by more than one peptide (missed cleavages) and some peptides represent more than one site (co-elution or ambiguous localisation) (see Experimental Procedures). Mean coverage of Set2p by proteolytic cleavage with trypsin was 79.9%. The mean phosphorylation of four biological replicates is shown, and one technical replicate was performed. Statistical analysis was performed using an unpaired *t*-test with multiple comparisons (two stage step-up method of Benjamini, Krieger and Yekutieli, 1% FDR) to compare the relative abundance of Set2p phosphopeptides in the 1 M NaCl treated cells to those in the unstressed cells.

Rph1p has at least 36 phosphorylated residues (1) making it a likely integrator of many kinase-mediated signaling events (Fig. 1). Interestingly, nine phosphosites have an adjacent proline, being found in an S/T-P motif. To identify sites of regulatory potential, we over-expressed and purified Rph1p from *S. cerevisiae* cells which had been treated with 1 M NaCl or 15 mM caffeine for 15 min. We used salt stress as its cellular response is well-characterized and involves highly conserved signaling pathways, and caffeine as it is a target of rapamycin (TOR) inhibitor that mimics many of the cellular effects of nutrient limitation—a process in which Rph1p has a regulatory role (9, 18, 25). To measure phosphorylation levels, purified Rph1p was digested using trypsin and peptides analyzed by liquid chromatography tandem mass spectrometry (LC-MS/MS). Across all conditions, 14 phosphopeptides were consistently quantifiable. These contained 20 of the 36 previously reported phosphosites (1). We could specifically localize 15 sites via MS2 (Fig. 2*A*, Supplemental Fig. S1, and Supplemental Table S1), including seven proline-adjacent sites. The remaining 16 previously reported Rph1p phosphosites were not seen in our methods; they were either discovered using alternative proteases or MS/MS fragmentation techniques, may only be phosphorylated under a particular growth condition, or may reflect mis-assignment in the literature due to non-specific localization. We also quantified phosphorylation at three sites not previously reported in (1) – S72, T580 and T669. One (S72) is of very low relative phosphorylation, making it difficult to detect, and the others (T580 and T669) are present in multi-site phosphopeptides wherein assignment can be difficult.

On 1 M NaCl treatment, seven Rph1p phosphopeptides had significantly increased phosphorylation (Fig. 2*A*). Meanwhile, two phosphopeptides had significantly increased phosphorylation upon 15 mM caffeine treatment (Fig. 2*A*); these also increased upon salt stress. These peptides corresponded to an increase in Rph1p phosphorylation at 11 and four sites, respectively. Inspection of the linear sequences carrying upregulated Rph1p phosphosites in salt stress showed enrichment for proline-adjacent motifs (*i.e.* pS-P) (Fig. 2*B*). Except for S72, all proline-adjacent sites measured by LC-MS/MS in salt stress showed a significant increase in relative phosphorylation. This may be because phosphorylation at this site is constitutive and not regulatory, and therefore necessary for Rph1p function. S72 is in a more structured region of Rph1p, between its JmjN and JmjC domains (PDB: 3OPT), unlike the other stress-responsive, proline-adjacent sites. Together, our results show that Rph1p is hyperphosphorylated in response to 1 M NaCl, particularly at S-P sites.

Given the significant upregulation of Rph1p phosphosites in response to salt stress, we investigated whether *S. cerevisiae* Set2p, which methylates H3K36, also showed changes in phosphorylation. Set2p was over-expressed and purified from *S. cerevisiae* cells which had been treated with 1 M NaCl for 15 min. Set2p has 18 reported phosphorylated residues (1), including three proline-adjacent sites. We could consistently quantify seven tryptic phosphopeptides which covered 13 of those sites (including two proline-adjacent). Seven of these sites could be specifically localised *via* MS2 (Fig. 2*C*, Supplemental Fig. S2, and Supplemental Table S2). From those, we found a significant increase in relative phosphorylation at only one phosphopeptide, which contained the S522 proline-adjacent site (Fig. 2*C*). Our results indicate that Set2p phosphosites are less salt stress-responsive than Rph1p phosphosites, suggesting that any salt stress-associated modulation of H3K36 methylation by upstream phosphorylation is largely mediated by demethylases.

### Loss of Rph1p Phosphorylation Affects Cell Growth Upon Salt Stress

**Fig. 3:**
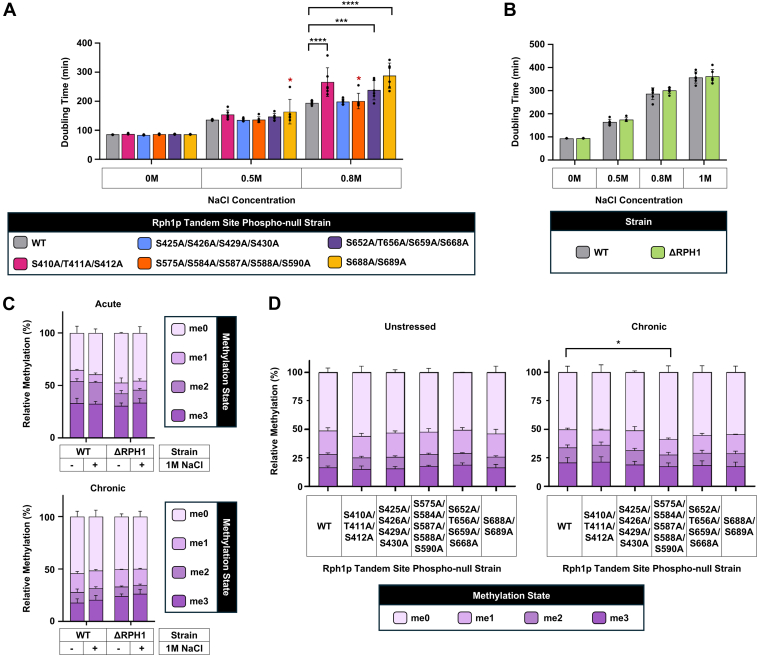
**Some Rph1p tandem site phosphonull mutants show slow growth on salt stress but this is not associated with gross changes in H3K36 methylation**. *A*, doubling time of Rph1p tandem site phosphonull mutant strains upon increasing concentrations of NaCl in synthetic complete media. The doubling time of six biological replicates is shown, and one technical replicate was performed. Outliers are denoted with a *red asterisk* (∗) and were determined by applying the ROUT method to each set of biological replicates (Q = 1%). Statistical analysis (with outliers removed) was performed using a two-way ANOVA with multiple comparisons (Dunnet statistical hypothesis testing, adjusted *p* value < 0.05) to compare the doubling time of the Rph1p tandem site phosphonull mutant strains to the wildtype. *B*, doubling time of ΔRPH1 strain upon increasing concentration of NaCl in synthetic complete media. The doubling time of six biological replicates is shown, and one technical replicate was performed. Statistical analysis was performed using an unpaired *t*-test with multiple comparisons (two stage step-up method of Benjamini, Krieger and Yekutieli, 1% FDR) to compare the doubling time of the ΔRPH1 strain to the wildtype. *C*, relative change in gross methylation at H3K36 of the wildtype and ΔRPH1 strain in the presence of zero, acute or chronic 1 M NaCl. Relative methylation state was determined using LC-MS/MS by measuring the amount of the methylpeptide (KSAPSTGGVKKPHR) compared to the total amount of the unmethylated peptide and all other differentially methylated versions (see Experimental Procedures). The mean relative methylation of three biological replicates is shown, and one technical replicate was performed. Statistical analysis was performed using an ordinal logistic model with proportional odds assumption (Supplemental Fig. S3). *D*, relative change in gross methylation at H3K36 of Rph1p tandem site phosphonull mutant strains in zero and chronic 1 M NaCl. Relative methylation was determined as described above. The mean relative methylation of three biological replicates is shown, and one technical replicate was performed. Statistical analysis was performed as described above (Supplemental Fig. S4).

To functionally investigate Rph1p phosphosites, we generated a library of phosphonull mutants, whereby phosphorylated residues were genomically mutated to alanine—making them unphosphorylatable. Phosphosites adjacent or near-adjacent in primary sequence were mutated in tandem within the same strain. In total, five strains were generated which corresponded to 18 phosphonull mutations (Fig. 3*A*); these covered all sites significantly salt stress-responsive, as per Figure 2*A*. To assess whether Rph1p phosphorylation was important for the salt stress response, Rph1p phosphonull mutant strains and the ΔRPH1 strain, were grown with zero, 0.5 M or 0.8 M NaCl, and doubling times were measured. At 0.8 M NaCl, three Rph1p tandem site phosphonull strains (i.e. Rph1p-S410A/T41p1A/S412A, Rph1p-S652A/T656A/S659A/S668A, and Rph1-S688A/S689A) had a significantly increased doubling time compared to the wildtype (Fig. 3*A*). This indicates that some or all of these sites are important for cell growth during salt stress. Rph1p phosphonull strains showed no significant change in doubling time compared to the wildtype in 0.5 M NaCl (Fig. 3*A*). Interestingly, the ΔRPH1 strain also showed no significant change in doubling time compared to the wildtype upon increasing concentrations of NaCl (Fig. 3*B*). Consequently, phosphorylation at some Rph1p sites is important for the response to salt stress yet a complete lack of Rph1p is not detrimental to the cell under the same conditions.

### Acute or Chronic Salt Stress Does Not Affect Gross Methylation Levels of H3K36

Rph1p targets the di- and tri-methylated form of H3K36 and is the only H3K36me3 demethylase in *S. cerevisiae* (2, 3, 4, 5, 6, 26). Therefore, we investigated whether Rph1p affects gross methylation levels at H3K36 in salt stress. To do this, wildtype cells were subject to either acute or chronic 1 M NaCl and gross H3K36 methylation levels were measured using LC-MS/MS. The ΔRPH1 strain was used as a control and to observe any background changes in H3K36 methylation that were not Rph1p-dependent. We found no significant change in gross H3K36 methylation states for wildtype cells subject to acute or chronic salt stress (Fig. 3*C* and Supplemental Fig. S3). Similarly, no significant changes were observed for the ΔRPH1 strain, suggesting that gross changes in H3K36 methylation do not occur in high salt stress in an Rph1p-dependent or independent manner. Together, this suggests that Rph1p may target specific regions of chromatin in response to high salt stress.

### Rph1p Tandem Site Phosphonull Mutants Weakly Affect H3K36 Gross Methylation Levels in Response to Chronic Salt Stress

To determine whether phosphorylation at specific sites on Rph1p affects gross methylation states in salt stress, the H3K36 methylation levels in the Rph1p tandem site phosphonull mutant strains were compared to the wildtype in chronic 1 M NaCl. In almost all cases, H3K36 methylation levels were the same between the Rph1p mutant strains and the wildtype when grown in either the presence or absence of high salt stress (Fig. 3*D* and Supplemental Fig. S4). The exception was the Rph1p-S575A/S584A/S587A/S588A/S590A strain, which showed a small but significant decrease in H3K36 methylation compared to the wildtype in the presence of chronic 1 M NaCl. These results indicate that salt stress-responsive phosphosites on Rph1p predominantly do not regulate gross changes in H3K36 methylation but may be responsible for targeted changes in chromatin.

### Loss of Rph1p Phosphorylation at Specific sites Affects Cell Growth upon Salt Stress

**Fig. 4:**
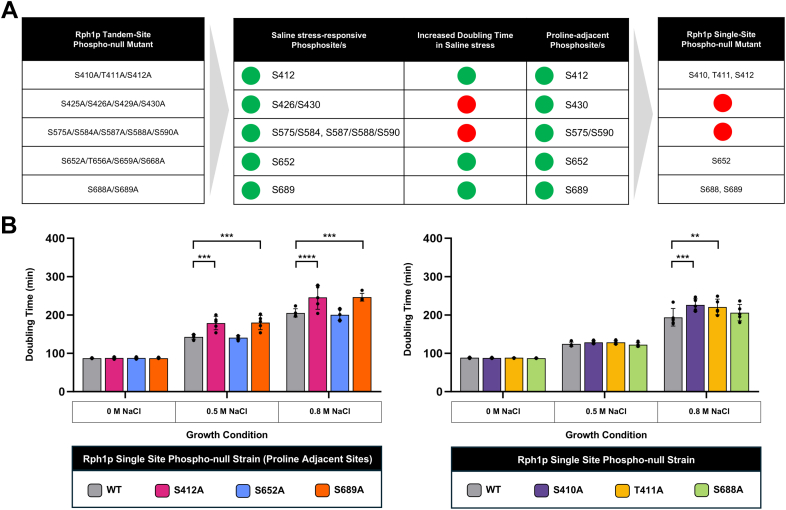
**Some Rph1p single site phosphonull mutants grow slower upon salt stress**. *A*, systematic approach to Rph1p phosphosite selection for single site mutagenesis. Sites that were salt stress-responsive and proline-adjacent, where the equivalent Rph1p tandem site phosphonull mutant strain showed increased doubling time in salt stress, were chosen for single site mutagenesis (*i.e.* S412, S652 and S689). S410, T411 and S688 were also mutated as they are adjacent to S412 and S689. *Green* indicates candidate sites and *red* indicates sites that were eliminated from selection. *B*, doubling time of Rph1p single site phosphonull strains upon increasing concentrations of NaCl in synthetic complete media. Proline-adjacent Rph1p single site phosphonull mutants are shown on the *left*, whilst the remaining mutants are shown on the *right*. The doubling time of five biological replicates is shown, and one technical replicate was performed. Statistical analysis was performed using a two-way ANOVA with multiple comparisons (Dunnet statistical hypothesis testing, adjusted *p*-value < 0.05) to compare the doubling time of the Rph1p single site phosphonull mutant strains to the wildtype.

To determine exactly which phosphosites are important for the cellular response to salt stress, we generated six single site Rph1p phosphonull mutant strains (Fig. 4*A*). Three of these sites – S412, S652 and S689 – were chosen because they were salt stress-responsive, proline-adjacent and their respective tandem site phosphonull mutant showed increased doubling time upon salt stress. Due to proximity in primary sequence, we also generated individual phosphonull mutants of S410, T411 and S688. We performed liquid growth assays on all six strains in the presence of zero, 0.5 M or 0.8 M NaCl. Two of the proline-adjacent mutant strains, Rph1p-S412A and Rph1p-S689A, had significantly increased doubling time compared to the wildtype in 0.5 M and 0.8 M NaCl, but showed no change in doubling time in the absence of NaCl (Fig. 4*B*). This suggests that phosphorylation at the S412 and S689 sites of Rph1p is important for the cellular response to salt stress. Interestingly, two of the non-proline-adjacent mutants, Rph1p-S410A and Rph1p-T411A, also showed a significant increase in doubling time compared to wildtype in 0.8 M NaCl (Fig. 4*C*). This suggests that these phosphosites may also contribute to salt stress-responsive regulation, highlighting that region of Rph1p to be of functional importance. Alternatively, it is possible that mutation of S410 or T411 affects the S412 kinase recognition motif, inhibiting phosphorylation and thus resulting in a similar phenotype. During manuscript revision, an unintended D155G substitution in the Rph1p-T411A strain was identified, which may contribute to the slow growth phenotype observed in Figure 4*B*. However, as the Rph1p-S410A and Rph1p-S412A strains also exhibited slow growth, loss of phosphorylation at T411 is still considered the most likely primary cause. Overall, our results show that phosphonull mutations of Rph1p at S410, T411, S412 and S689 result in decreased growth in the presence of salt stress, indicating that these sites are important for the yeast cell’s response to this condition.

### The Proteome of Rph1p-S412A and Rph1p-S689A Mutants is Not Significantly Different Compared to Wildtype in Chronic Salt Stress

**Fig. 5:**
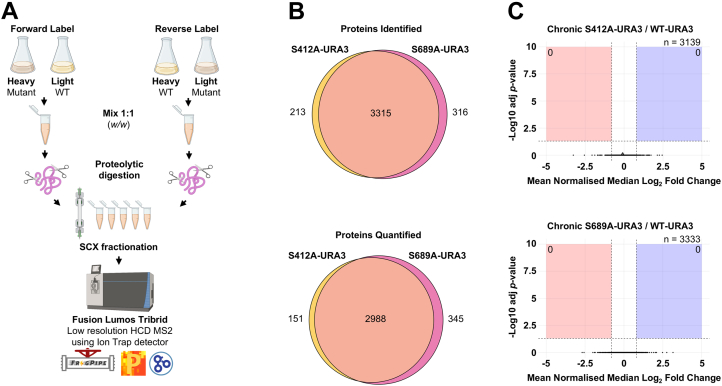
**Proteome profile of Rph1p-S412A and Rph1p-S689A strains compared to wildtype in response to chronic salt stress**. *A*, 2nSILAC workflow for proteomic analysis of Rph1p-S412A and Rph1p-S689A strains compared to wildtype. Mutant and wildtype strains were metabolically labelled with either heavy isotopes of arginine (^13^C_6_^15^N_4_/+ 10 Da) and lysine (^13^C_6_^15^N_2_/+ 8 Da) or light isotopes of arginine and lysine. Label swap experiments were conducted giving two biological replicates. Mixed lysates were subject to tryptic digestion and peptides were fractionated using strong cation exchange chromatography. LC-MS/MS was performed on the Fusion Lumos Tribrid using the Ion Trap mass analyser for MS2, with one technical replicate performed for each fraction. Data analysis was performed with the MSFragger SILAC3 workflow using IonQuant for MS1 quantification (see Experimental Procedures). Proteins identified by < 2 peptides in either replicate were removed, the reverse label for each experiment was multiplied by -1 and all resulting H/L ratios were normalised to control for mixing. The filtered normalised median Log2 H/L ratios for each biological replicate of the Rph1p-S412A and Rph1p-S689A experiments are in Supplemental Tables S6 and S7. Statistical analysis was performed using a one sample *t*-test where the mean normalised median Log2 fold change in abundance of each protein in the mutant strain relative to the wildtype was compared to the hypothetical value of 0 (no change) using a *q*-value < 0.05. *B*, venn diagrams of the number of proteins identified and quantified across both label swap replicates for the Rph1p-S412A and Rph1p-S689A experiments post filtering. *C*, volcano plots of the differentially abundant proteins in the Rph1p-S412A and Rph1-S689A strains compared to the wildtype strain in response to chronic salt stress. Proteins were classified as downregulated when they had a mean normalized median Log2 fold change ≤ -0.8 (*q*-value < 0.05) and upregulated when they had a mean normalized median Log2 fold change ≥ 0.8 (*q*-value < 0.05).

We investigated whether loss of Rph1p phosphorylation at salt-stress responsive sites S412 and S689, which is associated with slow growth in the salt stress response, impacts the proteome. As acute high salt stress causes a strong yet transient inhibition of protein synthesis (27) and Rph1p acts on chromatin, cells were subject to chronic stress to reflect the long-term cellular reprogramming and resumption of protein synthesis. To do this, we compared the Rph1p-S412A and Rph1p-S689A strains to the wildtype when grown in 1 M NaCl. 2nSILAC (21, 22) was used, with off-line fractionation and 3 h LC-MS/MS gradients (Fig. 5*A*). A reciprocal label swap design was used to generate forward and reverse biological replicates for each experiment. The incorporation of heavy arginine and lysine in lysates was >99% for all heavy-labelled samples, and the mean heavy-labelled proline conversion rate was 6.3% (Supplemental Table S5). Of the ∼4000 *S. cerevisiae* proteins typically expressed during log phase (28), we quantified 3139 and 3333 proteins in the Rph1p-S412A and Rph1p-S689A experiments, respectively (Fig. 5*B*, Supplemental Tables S6, and S7). We did not identify any proteins that were differentially abundant (*q*-value <0.05) between the mutant and wildtype strains (Fig. 5*C*). This suggests that phosphorylation of Rph1p at S412 or S689 does not regulate the protein-coding genome, at least in the conditions examined here.

### Loss of Rph1p Phosphorylation at S689 Affects snoRNA Transcription in Chronic Salt Stress

**Fig. 6:**
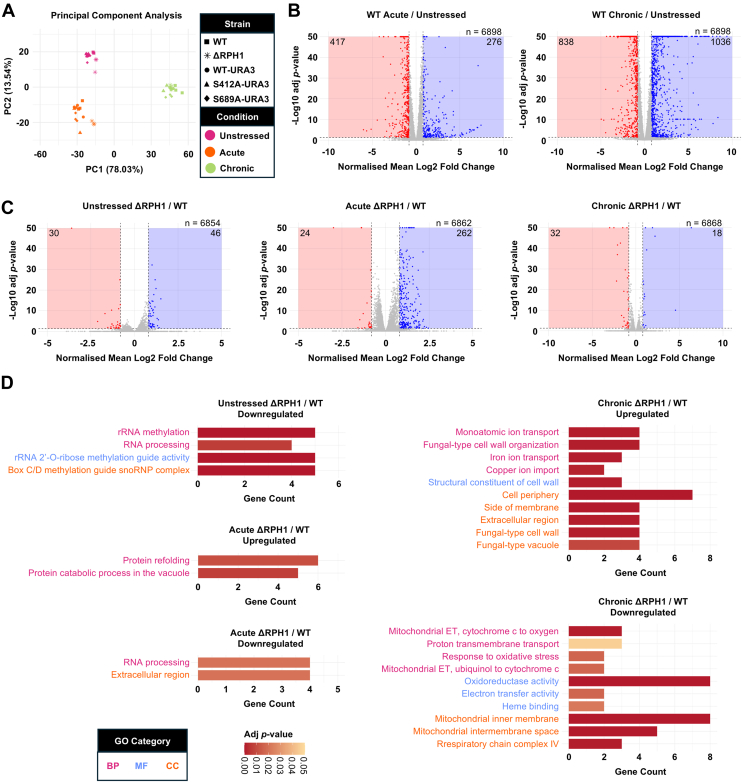
**Differentially expressed genes in wildtype in acute or chronic salt stress compared to no stress, and differentially expressed genes in ΔRPH1 compared to wildtype in no stress, acute or chronic salt stress.***A*, principal component analysis of all replicates across the five strains (WT, ΔRPH1, WT-URA3, RPH1-S412A-URA3 and RPH1-S689A-URA3) and three conditions (zero NaCl, acute 1 M NaCl and chronic 1 M NaCl). Genes where the summed read count across samples was ≤ 1 were removed and data was variance stabilised using the varianceStabilizingTransformation from DESeq2 (version 1.46.0) for exploratory analysis. *B*, volcano plots of differentially expressed genes in the wildtype strain in acute and chronic salt stress compared to no stress. The normalised mean Log2 fold change of three biological replicates is shown. Genes were classified as downregulated when they had a normalised mean Log2 fold change ≤ -0.8 (adjusted *p*-value < 0.05) and upregulated when they had a normalised mean Log2 fold change ≥ 0.8 (adjusted *p*-value < 0.05). *C*, volcano plots of differentially expressed genes in the ΔRPH1 strain compared to the wildtype strain in all conditions. The normalised mean Log2 fold change of three biological replicates is shown. Genes were classified as downregulated when they had a normalised mean Log2 fold change ≤ -0.8 (adjusted *p*-value < 0.05) and upregulated when they had a normalised mean Log2 fold change ≥ 0.8 (adjusted *p*-value < 0.05). *D*, significant GO terms of mRNA transcripts upregulated and downregulated in the ΔRPH1 strain compared to the wildtype strain in all conditions. The top 12 enriched GO terms by adjusted *p*-value are presented independent of GO category. Functional enrichment was performed using Enricher with quantified mRNA transcripts set as the background. Statistical analysis was performed using a hypergeometric test with Benjamini-Hochberg FDR correction (adjusted *p*-value < 0.05). BP, biological process; CC, cellular component; MF, molecular function.

As the proteome of our Rph1p-S412A and Rph1-S689A strains did not show significant change in chronic salt stress, but had clear growth phenotypes, we performed RNA-sequencing to investigate any changes in the transcriptome. We broadened our experiment to include both acute and chronic salt stress conditions, and examined this for the wildtype, Rph1p-S412A, Rph1p-S689A, and ΔRPH1 strains. Principle component analysis of resulting transcriptomes showed entirely clear separation via condition, and some separation of strains within each condition (Fig. 6*A*).

The transcriptional response of yeast to salt stress is well documented and varies greatly with strain, salt type, concentration and time (27, 29, 30, 31, 32, 33, 34). To benchmark our analysis, the transcriptional response of the wildtype strain to both acute and chronic 1 M NaCl compared to zero NaCl was first measured. Of the 6898 transcripts quantified, 276 were significantly upregulated and 417 were significantly downregulated in acute salt stress (Fig. 6*B*). A greater but different pattern of change was observed in chronic salt stress, with 1036 genes significantly upregulated and 838 genes significantly downregulated (Fig. 6*B*). This is consistent with reports that severe NaCl stress delays gene induction compared to mild NaCl stress (27, 32, 35). In line with previous studies (27, 34), many upregulated genes encoded proteins of unknown function, with 42% in acute and 21% in chronic salt stress. Also consistent with previous studies (27, 29, 30, 32, 33, 34, 35), genes involved in glycerol metabolism were upregulated in salt stress: GPD1 increased 2-fold (acute) and 10-fold (chronic), GPP2 increased 10-fold (chronic) and DAK1 increased 1.4-fold (acute) and 8-fold (chronic). STL1, encoding a glycerol proton symporter, was also upregulated in acute (6-fold) and chronic (672-fold) salt stress, agreeing with prior findings (27, 29, 32, 33, 34, 35). Functional enrichment (Gene Ontology, GO) analysis of upregulated genes in acute salt stress showed significant enrichment for translation regulation, whereas downregulated genes were significantly enriched for GO terms related to rRNA modifications, signaling, and the cell cycle (Supplemental Fig. S6). These enrichments were expected, as salt stress causes strong yet transient inhibition of protein synthesis (27), transient cell cycle arrest (36, 37) and supresses some constitutive signaling pathways whilst inducing those that are stress-specific (31, 38). In chronic salt stress, upregulated genes showed significant functional enrichment for oxidoreductase activity, cell morphology/structure and carbohydrate metabolism, whereas downregulated genes were significantly enriched for ribosome biogenesis (Supplemental Fig. S6). This reflects the excess production of reactive oxygen species (ROS) (39) and changes in cell morphology/structure that occur upon salt stress (40, 41, 42, 43).

Next, the ΔRPH1 strain was compared to the wildtype to measure how a complete loss of Rph1p function affects the transcriptome in zero, acute and chronic 1 M NaCl. Compared to the wildtype, the ΔRPH1 strain showed 46, 262 and 18 significantly upregulated genes when grown in zero, acute and chronic 1 M NaCl, respectively (Fig. 6*C*). Most upregulated genes in response to acute and chronic 1 M NaCl were stress-specific (Supplemental Fig. S7). Consistent with its known function (7, 8, 9, 10, 11, 12, 13), this suggests that Rph1p acts primarily as a transcriptional repressor and is important in de-repressing stress-responsive genes in acute salt stress and to a lesser extent, chronic salt stress. Interestingly, in the absence of salt stress there were no GO terms enriched for upregulated genes. By contrast, genes upregulated in response to acute salt stress showed significant functional enrichment for protein refolding and degradation. Several of these genes are known to be expressed under osmotic or oxidative stress (MTL1, GID10, PAI3, SSA4, and HSP78) or involved in degradation of gluconeogenic genes (VID24 and SSA1). In chronic salt stress, upregulated genes showed significant enrichment for cell wall structure/morphology and ion transport. Several of these cell wall genes encoded mannoproteins (FIT3, DAN1, TIP1, TIR1, and TIR3), three belonging to the Srp1p/Tip1p family (TIP1, TIR1, and TIR3) and one (DAN1) which was similar to TIR1-4. The limited overall change in the ΔRPH1 transcriptome in chronic salt stress compared to the wildtype, reflects the absence of an ΔRPH1 growth phenotype in increasing NaCl concentrations (Fig. 3*B*).

Interestingly, the number of significantly downregulated genes (∼30) in the ΔRPH1 compared to wildtype was similar across all conditions (Fig. 6*C*). Despite this, most downregulated genes upon knock out of RPH1 were condition-specific (Supplemental Fig. S7). In the absence of salt stress, downregulated genes in the ΔRPH1 showed significant functional enrichments related to C/D box snoRNP complexes and rRNA methylation (Fig. 6*D*). In particular, five snoRNAs (SNR54, SNR59, SNR67, SNR38, and SNR78) were downregulated, four of which guide 2′-O-ribose methylation to the ribosome large subunit (LSU) rRNA, and one to the small subunit (SSU) rRNA. Similarly, functional enrichment analysis on downregulated genes in acute salt stress showed significant enrichment for RNA processing (Fig. 6*D*), including three snoRNAs (SNR47, SNR57, and SNR67) and RPM2, a subunit of mitochondrial ribonuclease P which generates mature tRNA. In chronic salt stress, downregulated genes showed significant enrichment for oxidative phosphorylation and oxidoreductase activity (Fig. 6*D*).

**Fig. 7:**
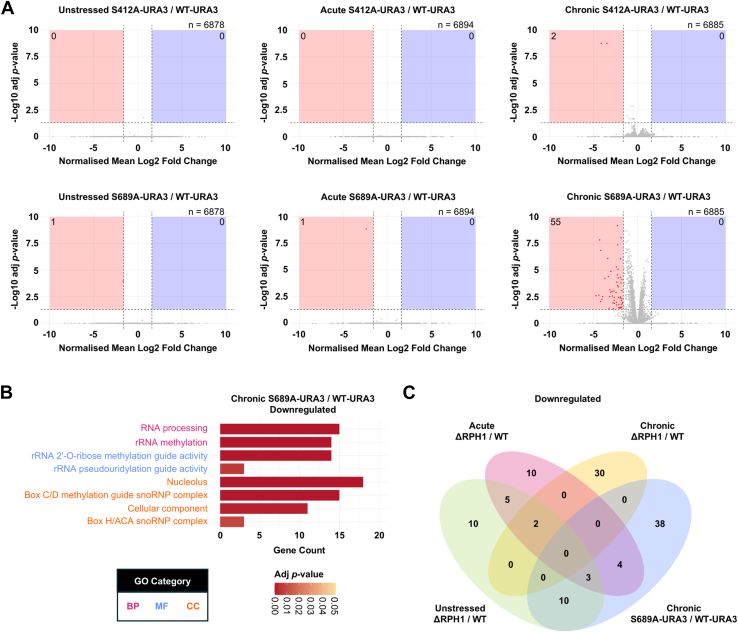
**Differentially expressed genes in Rph1p-S412A and Rph1p-S689A strains compared to wildtype upon acute and chronic salt stress.***A*, volcano plots of differentially expressed genes in the Rph1p-S412A and Rph1-S689A strains compared to the wildtype strain in all conditions. The normalised mean Log2 fold change of three biological replicates is shown. Genes were classified as downregulated when they had a normalized mean Log2 fold change ≤−0.8 (adjusted *p*-value <0.05) and upregulated when they had a normalized mean Log2 fold change ≥0.8 (adjusted *p*-value <0.05). *B*, significant shared GO terms of mRNA transcripts downregulated in the Rph1p-S689A strain compared to the wildtype strain in chronic salt stress. The top 12 enriched GO terms by adjusted *p*-value are presented independent of GO category. Functional enrichment and statistical analysis were performed as described above. *C*, four-way venn diagram of significantly downregulated genes in ΔRPH1 compared to wildtype in zero, acute and chronic 1 M NaCl and in the Rph1p-S689A strain compared to wildtype in chronic 1 M NaCl. BP, biological process; CC, cellular component; MF, molecular function.

We examined how loss of Rph1p phosphorylation at S412 and S689 affects the transcriptome in zero, acute and chronic 1 M NaCl when compared to the wildtype. A secondary question was whether mutagenesis of specific phosphosites elicits the same or a different transcriptional response. Remarkably, the only strong transcriptional change was seen in the Rph1p-S689A strain in chronic salt stress, which showed significant downregulation of 55 genes compared to the wildtype (Fig. 7*A* and Supplemental Table S8). These had significant functional enrichment for GO terms related to snoRNP complexes and rRNA modifications (Fig. 7*B*). This was primarily due to the downregulation of 18 snoRNA genes (Supplemental Table S8), of which SNR65 was the most downregulated (4.3-fold decrease). Of the 18 downregulated snoRNAs, 15 guide 2′-O-ribose methylation, with 12 to the ribosome LSU rRNA and three to the ribosome SSU rRNA, and a further three snoRNAs guide pseudouridinylation to the ribosome LSU rRNA (Supplemental Table S8). Interestingly, 13 of the downregulated genes, including three snoRNAs (SNR38, SNR59, and SNR67) were also downregulated in the ΔRPH1 in the absence of NaCl, whereas seven genes (including SNR49, SNR57, SNR67) were downregulated in the ΔRPH1 in acute salt stress (Fig. 7*C*). However, there were no downregulated genes in common between the Rph1p-S689A strain and ΔRPH1 strain in chronic salt stress (Fig. 7*C*). Therefore, loss of phosphorylation at Rph1p-S689 is not equivalent to loss of Rph1p function in chronic salt stress, however, it appears to partially mimic the effect of RPH1 knock out in zero and acute 1 M NaCl. This indicates that phosphorylation at S689 selectively affects Rph1p targeting during the chronic salt stress response. Most of the other downregulated transcripts in the Rph1p-S689A strain compared to the wildtype in chronic salt stress were dubious open reading frames or encoded putative proteins of unknown function. In contrast to our findings with the Rph1p-S689A mutant, few significant changes were observed in transcripts of Rph1p-S412A mutant cells when compared to wildtype (Fig. 7*A*). In chronic 1 M NaCl, only two genes (ERR3 and SNO4) were significantly downregulated. ERR3 encodes phosphopyruvate hydratase, which is important in glycolysis whereas SNO4 encodes a probable glyoxalase which is involved in protection against ROS.

## Discussion

Here, we show that the histone KDM Rph1p is extensively phosphorylated in acute high salt stress. Phosphonull mutations of Rph1p at the salt stress-responsive sites S410, T411, S412 and S689 impaired yeast cell growth in NaCl-supplemented media, indicating that phosphorylation at these sites is important for an effective salt stress response. Compared to wildtype, Rph1p-S412A and Rph1p-S689A phosphonull mutations did not change the proteomic response in chronic salt stress, suggesting that phosphorylation at these sites does not affect the chromatin of protein coding genes. However, transcriptomic analysis revealed a specific and marked downregulation of snoRNA genes associated with rRNA modifications in the Rph1p-S689A mutant in chronic salt stress, a response absent in the Rph1p-S412A mutant. Consistent with the limited transcriptional changes observed, gross H3K36 methylation levels were not markedly changed in Rph1p phosphosite mutants in a salt stress-dependent or -independent manner. This suggests that Rph1p phosphorylation at S689 has a targeted effect on transcription, rather than broadly altering chromatin state and H3K36 methylation. Together, our results suggest that individual phosphorylation sites on Rph1p can have distinct, stress condition-specific, effects on chromatin.

Our findings reveal that Rph1p′s role in the salt stress response is both condition- and phosphorylation-dependent. Loss of Rph1p phosphorylation at S689 caused pronounced repression of snoRNA genes in chronic salt stress. This is consistent with Rph1p′s reported occupancy at the rDNA locus (particularly 35S rDNA), at almost all ribosome protein genes, at 55 ribosome biogenesis genes, and at 62 of 77 genes encoding snoRNAs (13). Most of the snoRNA genes that changed in the Rph1p-S689A strain were also repressed in the wildtype in response to both acute and chronic salt stress. This indicates that their downregulation is a core component of the salt stress response and that this repression is further exacerbated in Rph1p-S689A mutant cells in chronic salt stress specifically. Given that Rph1p functions as a repressor in optimal conditions (7, 8, 9, 10, 11, 12, 13), this suggests that S689 phosphorylation facilitates de-repression of these snoRNA genes to maintain appropriate levels of expression in chronic salt stress. The overall lack of change of protein-coding genes in the Rph1p-S689A mutant was consistent with our proteomic analysis. This reveals an interesting role for Rph1p-S689 phosphorylation in non-coding RNA regulation, highlighting the functional importance of chromatin-RNA crosstalk during chronic salt stress.

In the Rph1-S689A strain, the majority of downregulated snoRNAs in chronic salt stress are responsible for guiding 2′-O-ribose methylation of the ribosomal rRNA, particularly the ribosome LSU rRNA (44, 45, 46, 47). Despite a few exceptions, the disruption of most individual snoRNAs impairing single or few modifications has been shown to have little or no impact on cell growth or translation efficiency in optimal conditions (44, 48). However, the deletion of individual C/D box snoRNAs can cause growth defects upon external perturbation (49). For example, knocking out of snoRNA SNR65 decreases growth rate upon ion stress via KCl treatment (49). Notably, we found that SNR65 was the most downregulated snoRNA in the Rph1-S689A mutant in chronic NaCl stress. More generally, the global loss of 2′-O-ribose methylation or pseudouridinylation sites results in severe phenotypes without majorly affecting rRNA processing, suggesting that the modification state of the ribosome is important for structure and translation accuracy (50, 51). Therefore, the combined downregulation of 18 snoRNAs observed in Rph1p-S689A cells in chronic salt stress may cause changes to ribosome function as part of long-term adaptation. This likely contributes to the impaired growth phenotype of the Rph1p-S689A mutant strain.

Many salt-stress responsive Rph1p phophosites identified here are consistent with those seen in large scale phosphoproteomics studies at different concentrations of NaCl (*e.g.* 0.4 M (52, 53, 54) and 0.5 M (55)) across different timepoints (*e.g.* 1 min (52), 5 min (53, 54), 10 min (55) and 20 min (54)). The large number of upregulated sites suggests Rph1p can integrate information from multiple kinase-mediated signaling pathways in response to salt stress. This might be expected, given NaCl induces both osmotic stress and cation toxicity, which secondarily induces the excess production of ROS (39, 56, 57, 58, 59, 60). Our findings align with studies showing that Rph1p becomes hyperphosphorylated in response to stressors such as rapamycin and DNA damage (13, 17). Many of our Rph1p salt-stress responsive phosphosites are found in proline-adjacent motifs, suggesting that proline-directed kinases are responsible for them. The high osmolarity glycerol (HOG) pathway is essential for the cell’s adaptation to high osmolarity (31). The cell wall integrity pathway is also activated by increased turgor pressure resulting from the accumulation of glycerol, which is dependent on the HOG pathway (38, 61). Key kinases belonging to these pathways, such as Hog1p and Slt2p, target S/T-P motifs (62) and, as such, may be capable of phosphorylating Rph1p sites under high salt stress. Moreover, salt stress is known to cause transient cell cycle arrest at the G1/G2 stage, which is at least partially regulated by Hog1p (36, 37). Cdk1p is considered the master regulator of the cell cycle (63, 64) and is a proline-directed kinase (62). It has been previously shown that the S430, S575, and S584 proline-adjacent sites in Rph1p, which we show to be salt stress-responsive, are likely downstream substrates of Cdk1p (65). Thus, other salt stress-responsive, proline-adjacent sites on Rph1p may be substrates of Cdk1p. While Rph1p may be regulated solely by kinases, it has been reported that Rph1p phosphorylation upon salt stress may also be regulated by the proline-directed protein phosphatase 2A through its regulatory subunit B (66).

Lack of Rph1p phosphorylation did not affect gross H3K36 methylation levels in response to high salt stress. This was expected as salt stress affects the expression of less than 10% of coding genes (≥2-fold) in wildtype yeast (27, 34, 35). Our transcriptomic analysis showed only a small number of genes changing in Rph1p-S412A and Rph1p-S689A mutant cells in chronic salt stress, which is also consistent with no gross change in H3K36 methylation. Studies of RPH1 deletion elsewhere also found no effect on gross H3K36 methylation levels, with only specific genes showing increased H3K36 trimethylation (3, 4, 6). Together, this suggests that salt-stress responsive phosphorylation of Rph1p likely regulates specific H3K36me targets in chromatin. This effect has been shown for several methylation enzymes in yeast (16, 67) and mammals (68, 69, 70). Nevertheless, it is possible that Rph1p has demethylase-independent functions which regulate the salt stress response in a phosphorylation-dependent manner. This is supported by the observation that some environmental stress response genes repressed by Rph1p do not require Rph1p-mediated demethylase activity (10, 25). Furthermore, it is known that JmjC demethylases in other organisms can act as transcription factors independently of their demethylase activity (71, 72, 73).

The acute salt stress response in ΔRPH1 was characterized by a substantial upregulation of genes compared to the wildtype. Many of these upregulated genes were also induced in the wildtype in response to acute salt stress, including GPD1 and STL1. This is consistent with Rph1p′s function as a negative regulator of stress-responsive genes under optimal conditions (7, 8, 9, 10, 11, 12, 13). However, in chronic salt stress, transcriptional changes between ΔRPH1 and wildtype were limited, with few upregulated genes and a slightly greater proportion of downregulated genes. This aligns with the absence of a growth phenotype and suggests cellular adaptation or the involvement of compensatory mechanisms. This effect has been documented for yeast histone demethylases in different conditions. For example, the Jhd1p-S44A phosphonull mutant is more sensitive to cold stress than JHD1 deletion (74), and the ΔRPH1 grows faster than wildtype in rapamycin and caffeine stress, whereas certain Rph1p phosphonull mutants are impaired (13). Compensation may be due to Jhd1p, which redundantly targets H3K36me2 (75), or with the paralog Gis1p, which shares homologous phosphosites, identical DNA-binding regions, and has overlapping gene targets, as well as cooperative and redundant functions in other stress responses (8, 9, 25). The slightly greater proportion of downregulated genes in ΔRPH1 in chronic salt stress also raises the possibility of a condition-dependent activator or stabiliser function, as observed for Rph1p post-diauxic shift (9, 25). Chronic salt stress causes upregulation of genes involved in oxidoreductase activity and the response to oxidative stress in the wildtype; however, these were repressed in the ΔRPH1. As both the diauxic shift and prolonged salt stress are accompanied by elevated levels of ROS (39, 76, 77), there may be some common Rph1p activator function. Taken together, these findings suggest that Rph1p is a multifunctional regulator whose role is condition-dependent and influenced by post-translational modifications.

In conclusion, our study systematically identified salt stress-responsive phosphorylation sites on Rph1p and showed that a subset of these sites is important for yeast cell growth in this condition. We identified S689 as a key Rph1p phosphorylation site that, in chronic salt stress, directly affects the regulation of snoRNA genes without affecting gross H3K36 methylation. Our work highlights the importance of Rph1p phosphorylation in modulating yeast cell adaptation to salt stress, offering valuable insights into stress-responsive regulatory mechanisms.

## Data Availability

All mass spectrometry data have been deposited in the ProteomeXchange Consortium via the PRIDE partner repository (78) with the dataset identifier PXD059945. XICs used for MS1 relative quantification in Rph1p and Set2p phosphorylation assays, and gross H3K36 methylation assays have been uploaded to Panorama, available at https://panoramaweb.org/vj4JGV.url. RNA-sequencing paired end reads are deposited in the NCBI Sequence Read Archive under BioProject ID PRJNA1306334.

## Supplemental Data

This article contains supplemental data (79, 80).

## Conflict of Interest

The authors declare that they do not have any conflicts of interest with the content of this article.
