# Supplementary material for: Proline-Adjacent Phosphosites on Saccharomyces cerevisiae Histone Demethylase Rph1p are Salt Stress Responsive and Important for Cell Growth Under Salt Stress
Source: Mol Cell Proteomics. 2025 Sep 8;24(10):101066. doi: 10.1016/j.mcpro.2025.101066 (PMC12546869; doi:10.1016/j.mcpro.2025.101066)
Supplement: Supplementary Material [file mmc1.docx]

Supplementary Material

Proline-adjacent phosphosites on *S. cerevisiae* histone demethylase Rph1p are salt stress responsive and important for cell growth under salt stress

Authors

Nicola M Karakatsanis^1,2^; Joshua J Hamey^1,2, ‡^; Marc R Wilkins^1,2, ‡^

Affiliations

^1^Systems Biology Initiative, School of Biotechnology & Biomolecular Sciences, UNSW Sydney, Australia

^2^ARC Centre of Excellence for the Mathematical Analysis of Cellular Systems, UNSW Sydney, Australia

‡Authors share co-senior authorship

Contents

**Figure S1.** MS2 spectra for localisation of phosphorylation within quantified Rph1p phosphopeptides

**Figure S2.** MS2 spectra for localisation of phosphorylation within quantified Set2p phosphopeptides

**Figure S3.** Ordinal logistic regression model of gross methylation levels at H3K36 of the wildtype and RPH1Δ strain in acute salt stress and chronic salt stress compared to no stress

**Figure S4.** Ordinal logistic regression model of gross methylation levels at H3K36 of the Rph1p phosphonull mutant strains compared to the wildtype strain in no stress and in chronic salt stress

**Figure S5.** Distribution of ratios for the 2nSILAC Rph1p-S412A and Rph1p-S689A experiments

**Figure S6.** Functional enrichment analysis of differentially expressed genes in the wildtype in acute and chronic salt stress compared to no stress

**Figure S7.** Venn diagrams of downregulated and upregulated genes in the ΔRPH1 compared to wildtype in no stress, acute salt stress and chronic salt stress

**Table S1.** Name, sequence and purpose of oligonucleotides used in this study

**Table S2.** Name and characteristics of plasmids used in this study

**Table S3.** Quantified Rph1p phosphopeptides

**Table S4.** Quantified Set2p phosphopeptides

**Table S5.** Efficiency of incorporation of heavy amino acid isotopes (arginine, lysine and proline) into yeast lysate based on peptide spectral matches

**Table S8.** Significantly (adjusted *p*-value < 0.05) downregulated (mean Log2 fold change ≤ 0.8) transcripts in the Rph1p-S689A strain compared to wildtype strain grown in chronic 1M NaCl


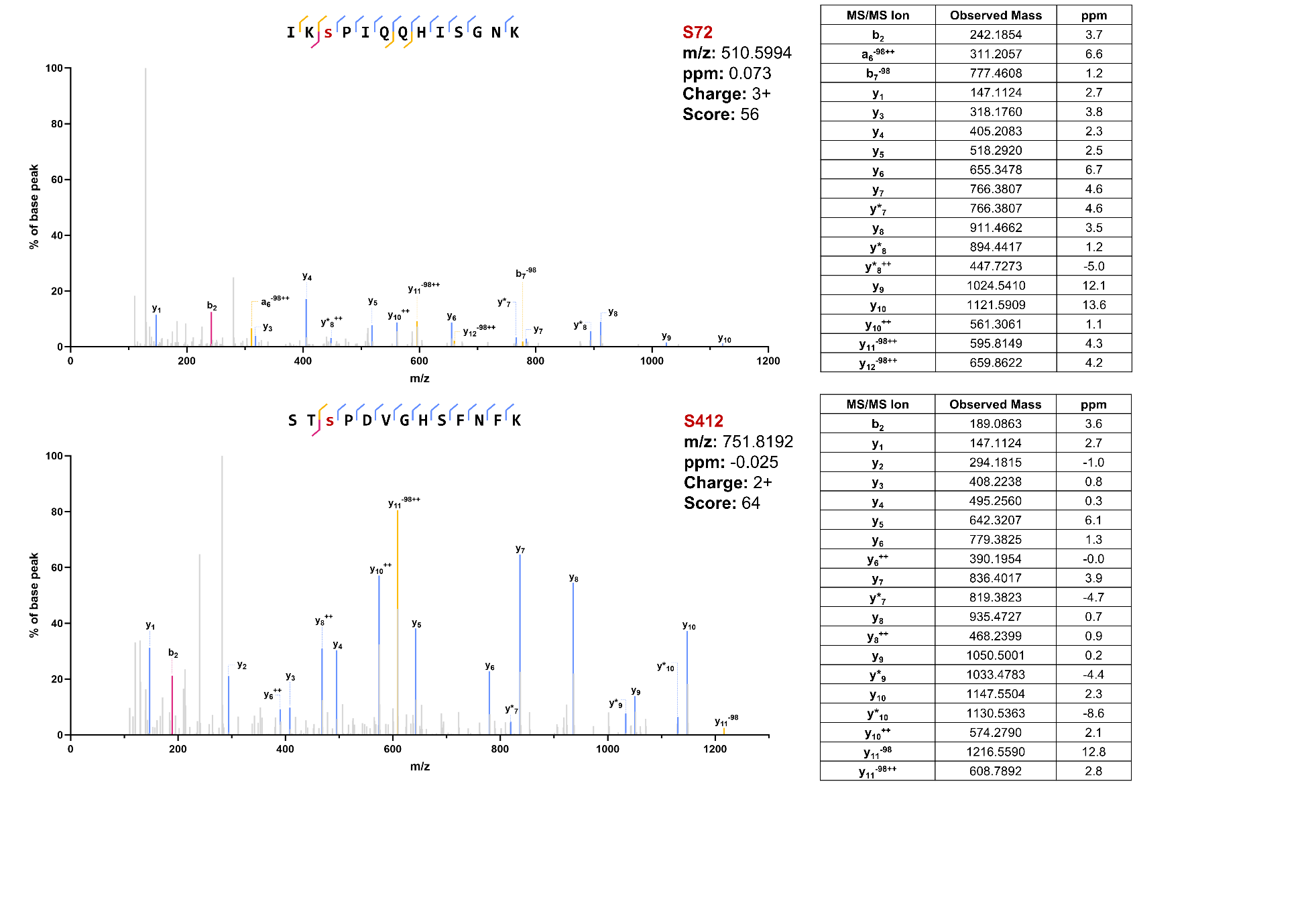


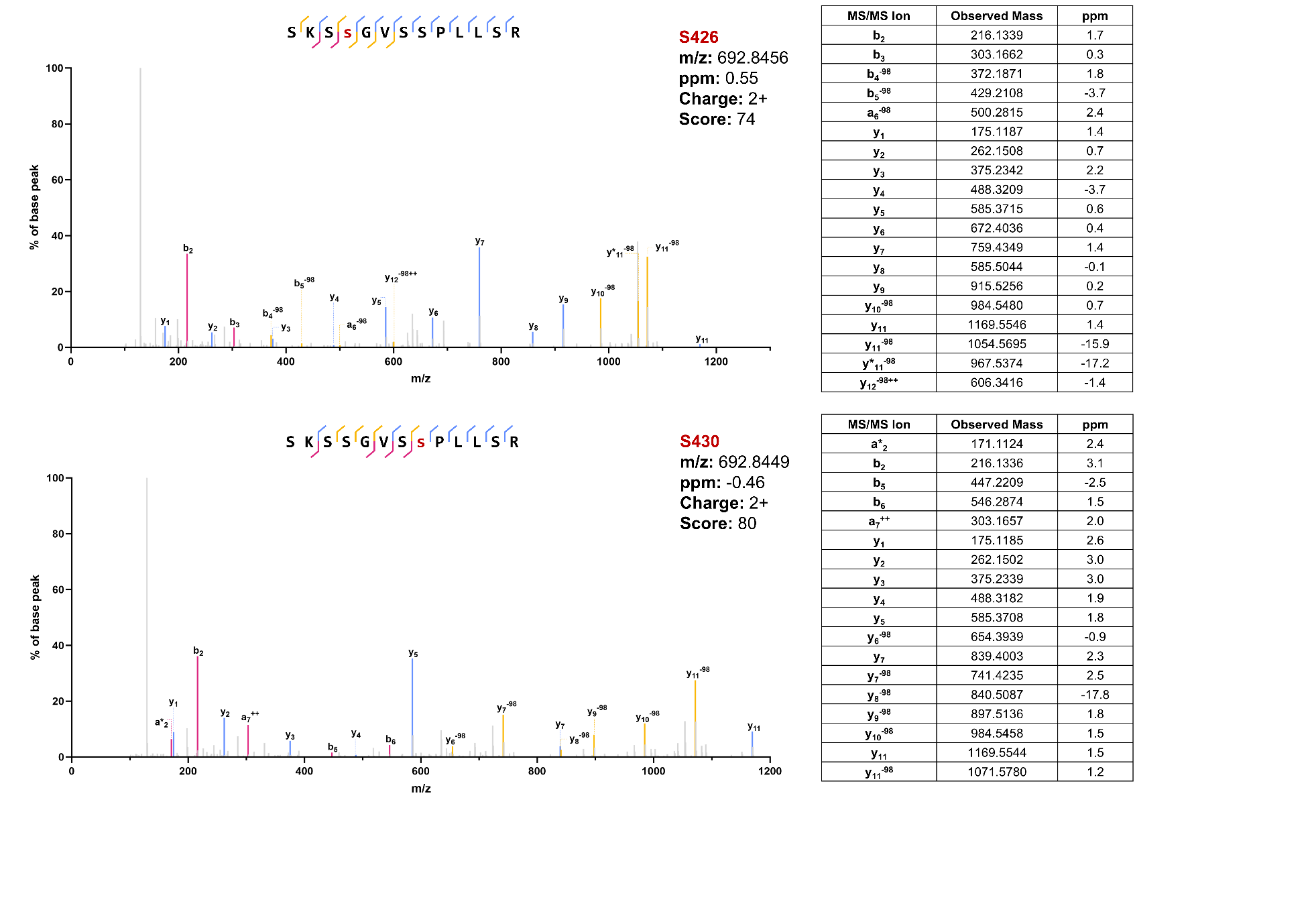


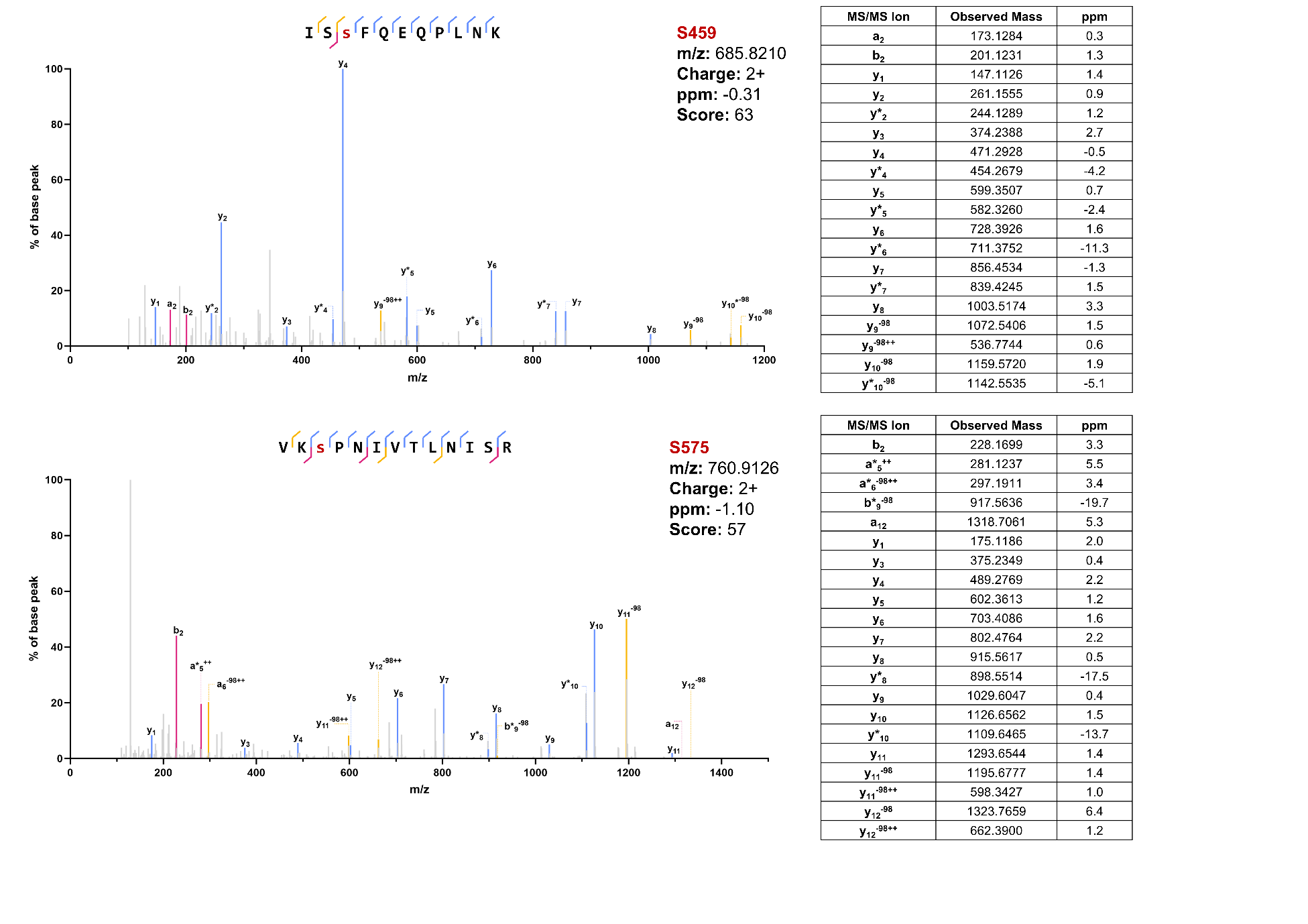


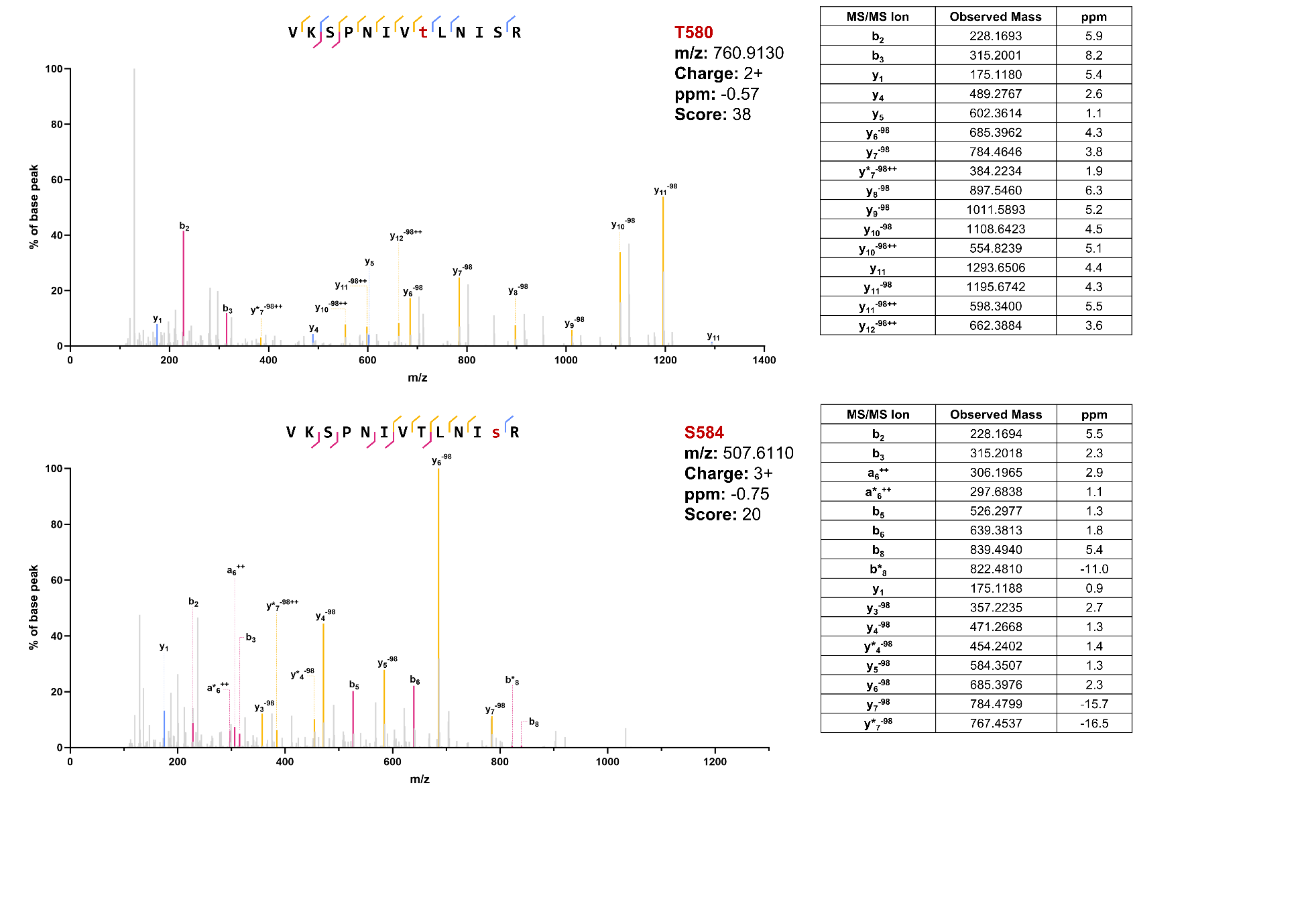


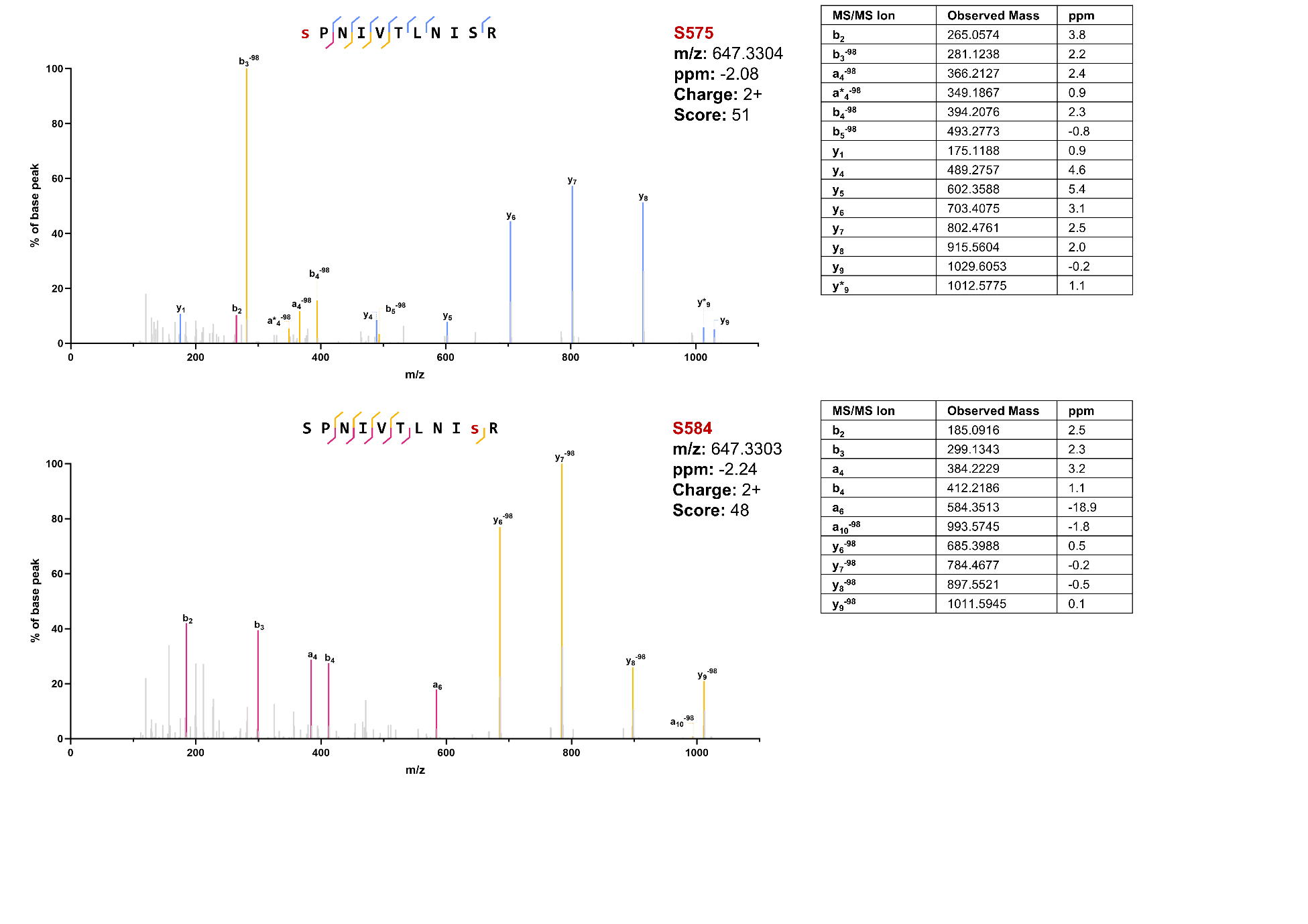


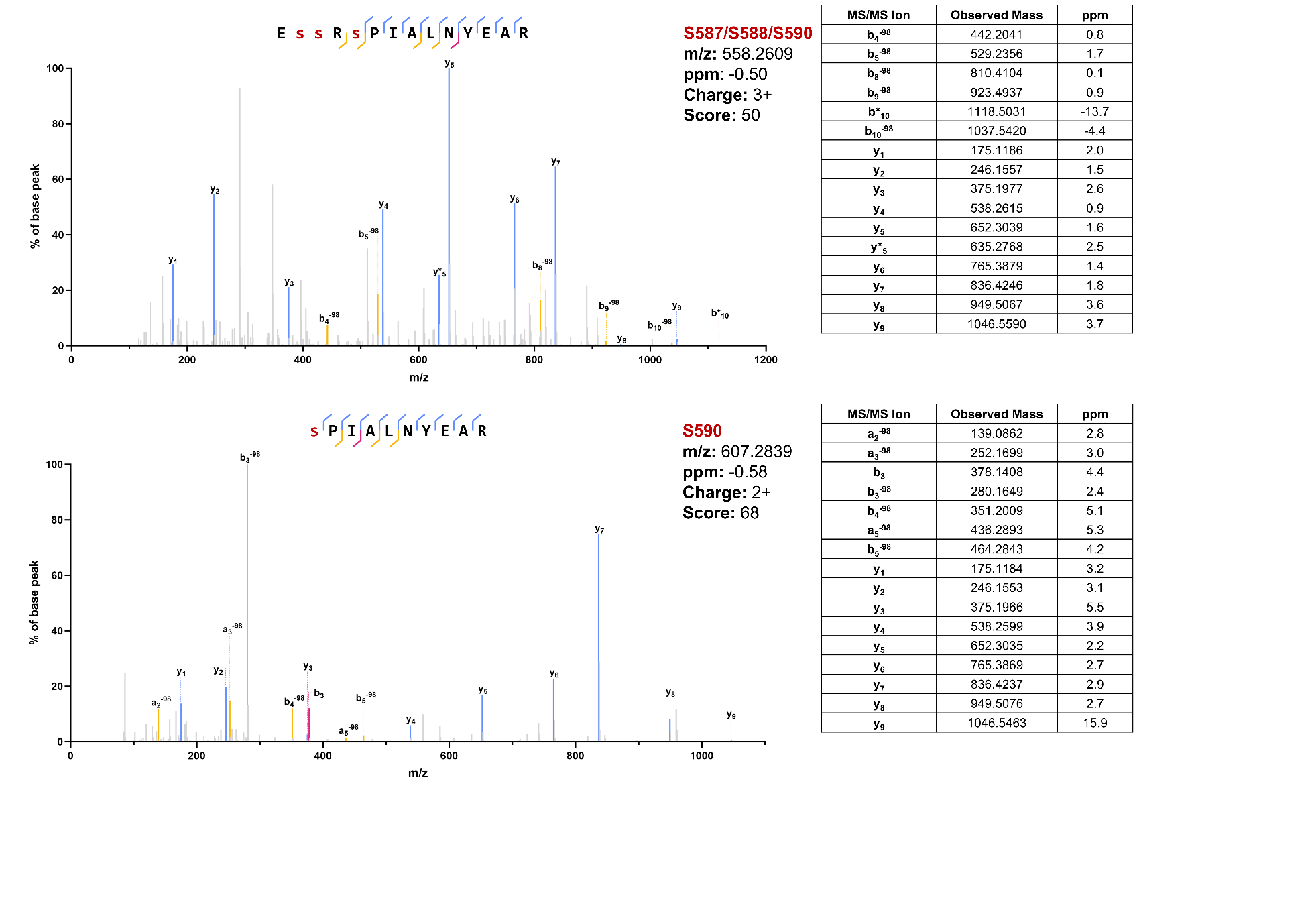


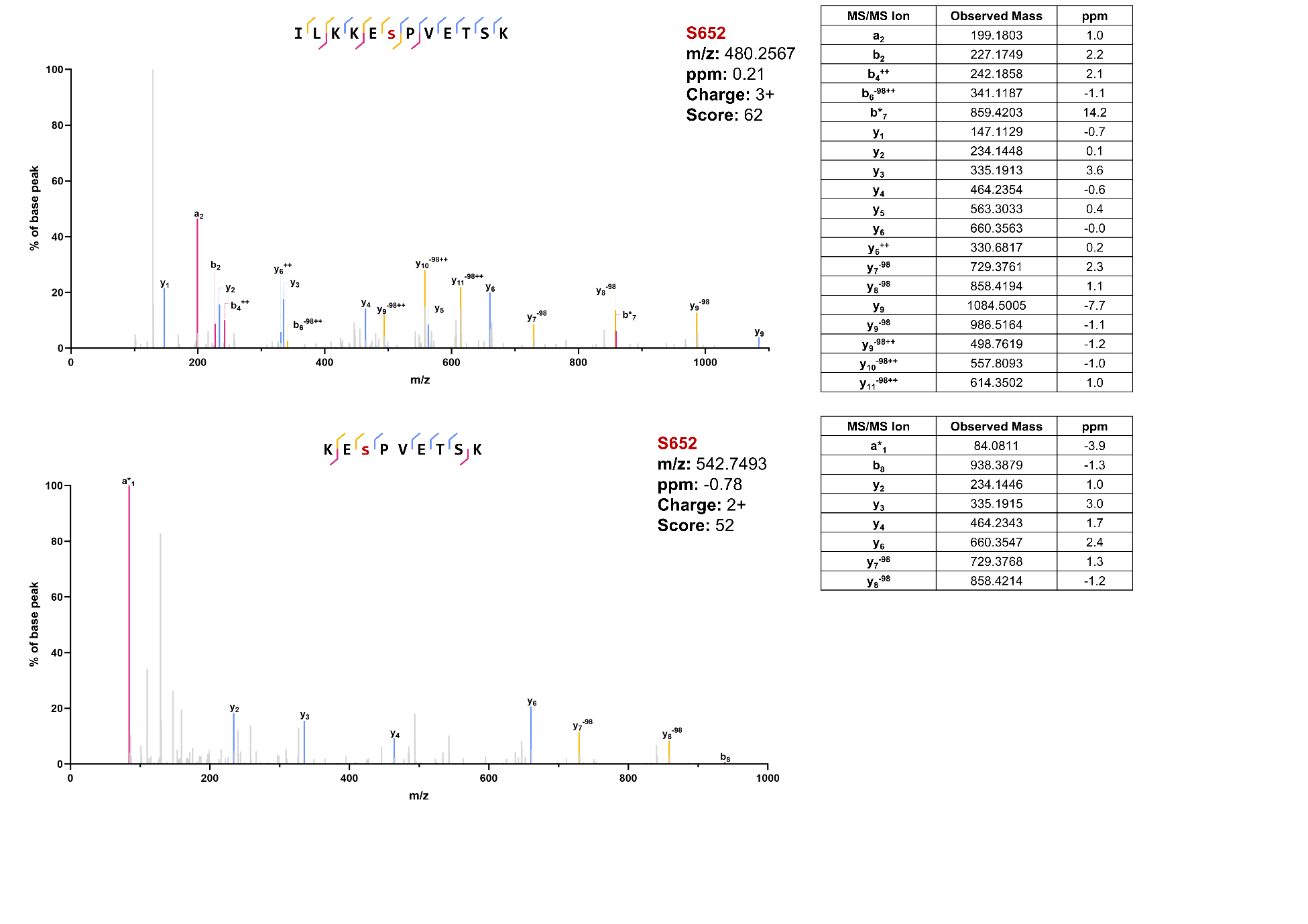


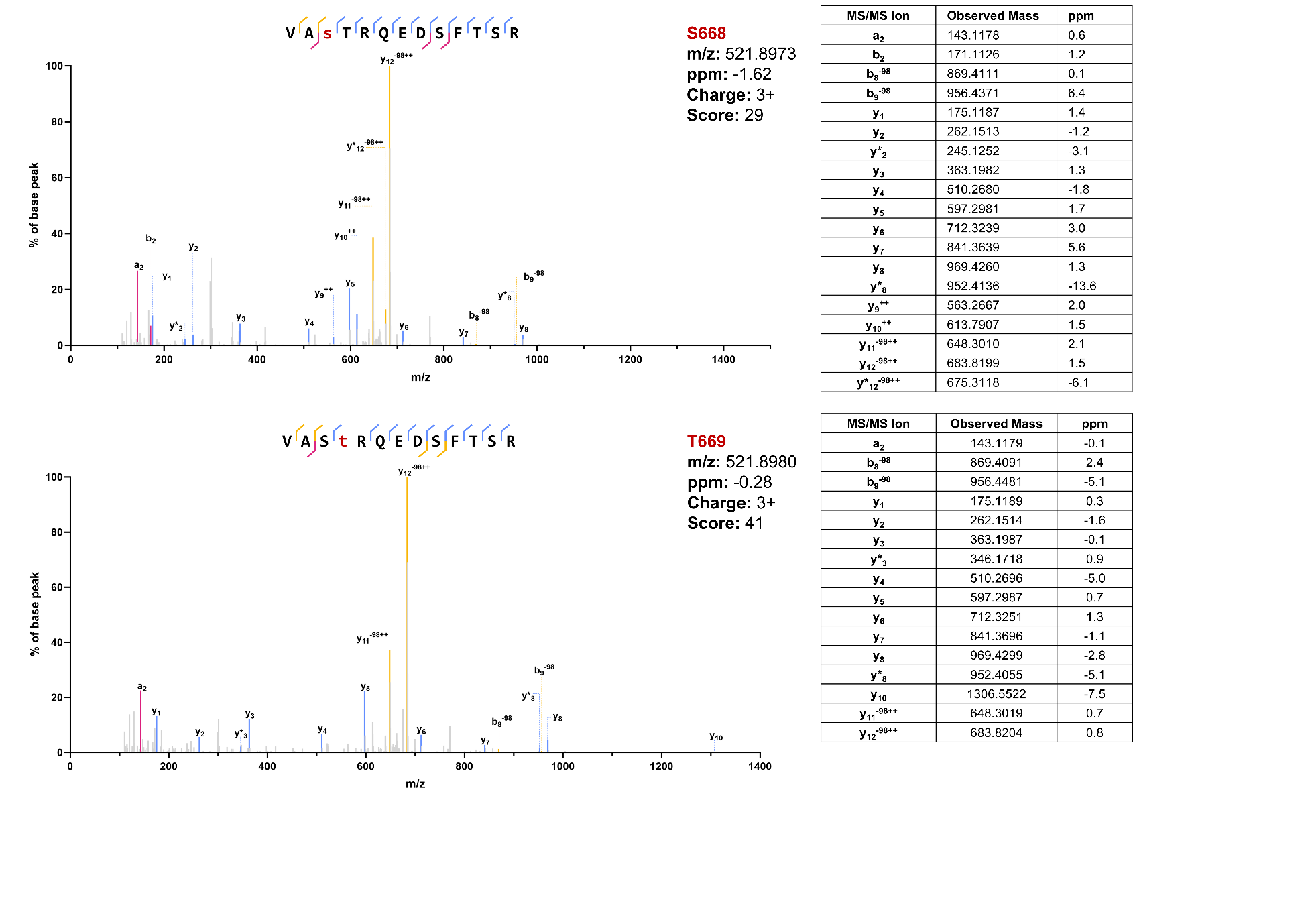


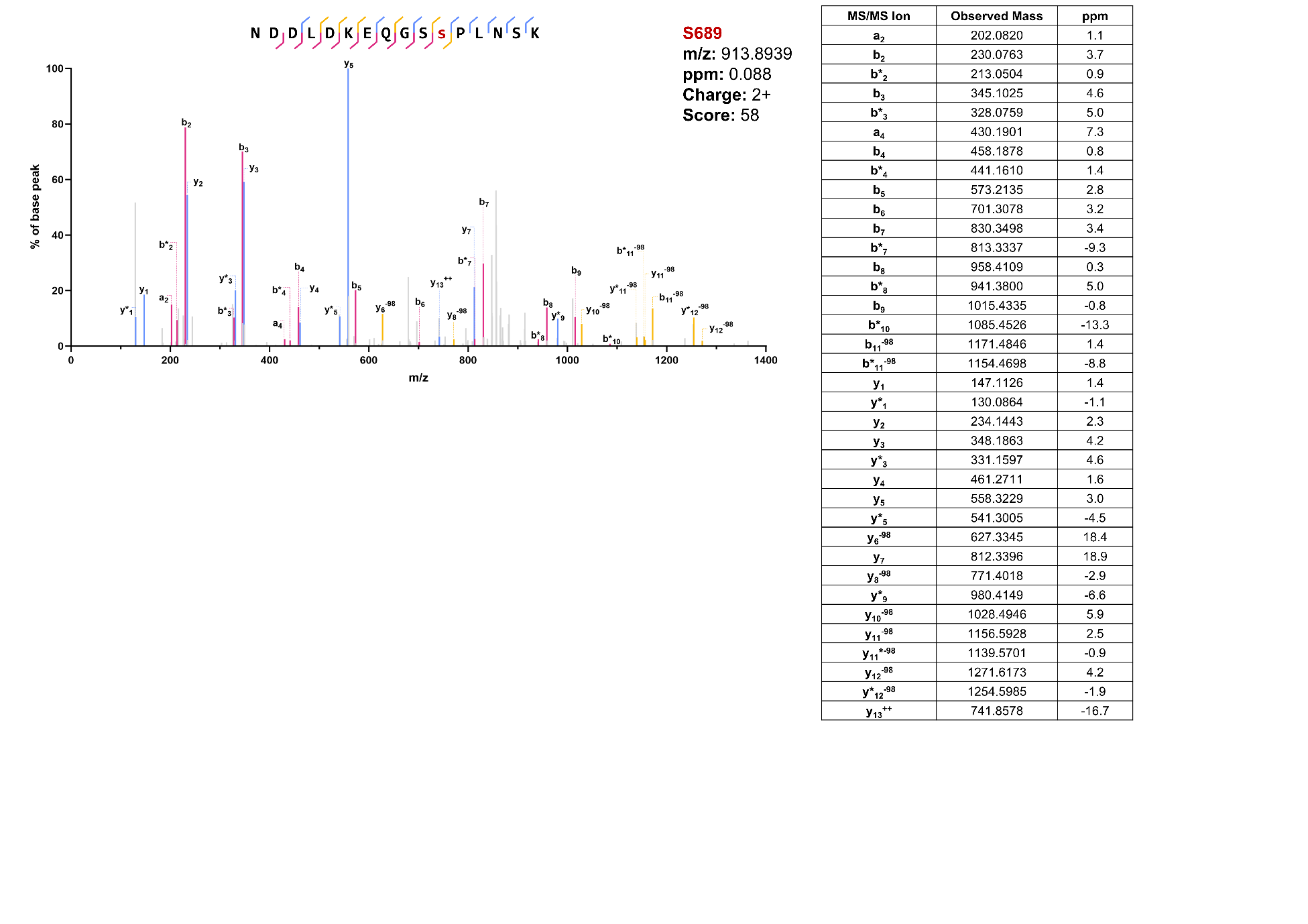


Figure S1. MS2 spectra for localisation of phosphorylation within quantified Rph1p phosphopeptides

*MS2 spectra used for localisation of phosphosites within quantified Rph1p phosphopeptides. b-ions are shown in pink, y-ions are shown in blue and b- and y-ions exhibiting a neutral loss of H_3_PO_4_ (-98) are shown in yellow. The localised phosphosite is shown in the peptide label as lowercase and burgundy. When there is more than one site indicated, this means the phosphosite could not be localised and any one singly phosphorylated version of the peptide may exist. MS2 spectra are replicated from the Mascot MS/MS Ions Search output, with only fragment ions used for scoring shown on each spectrum.*


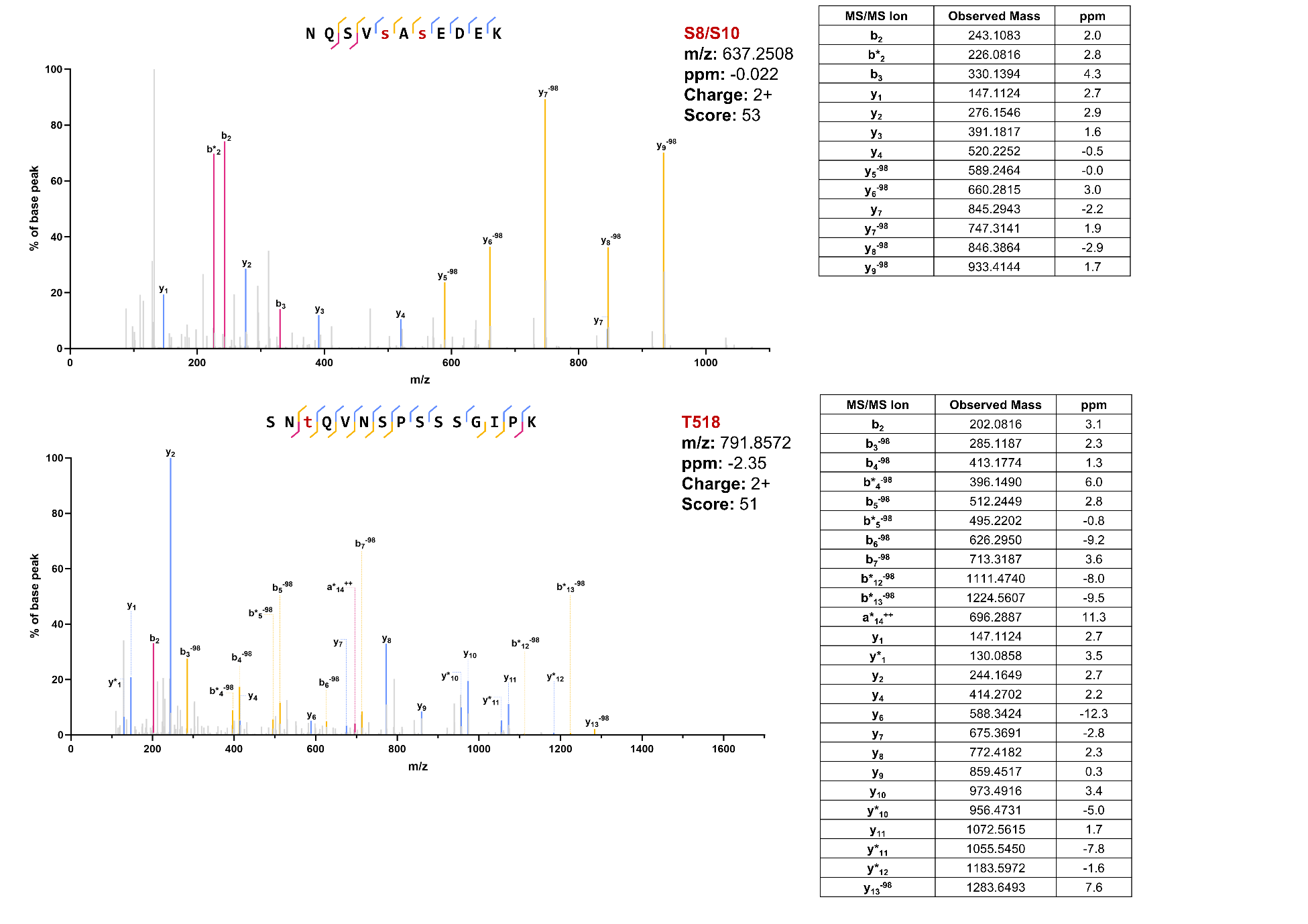


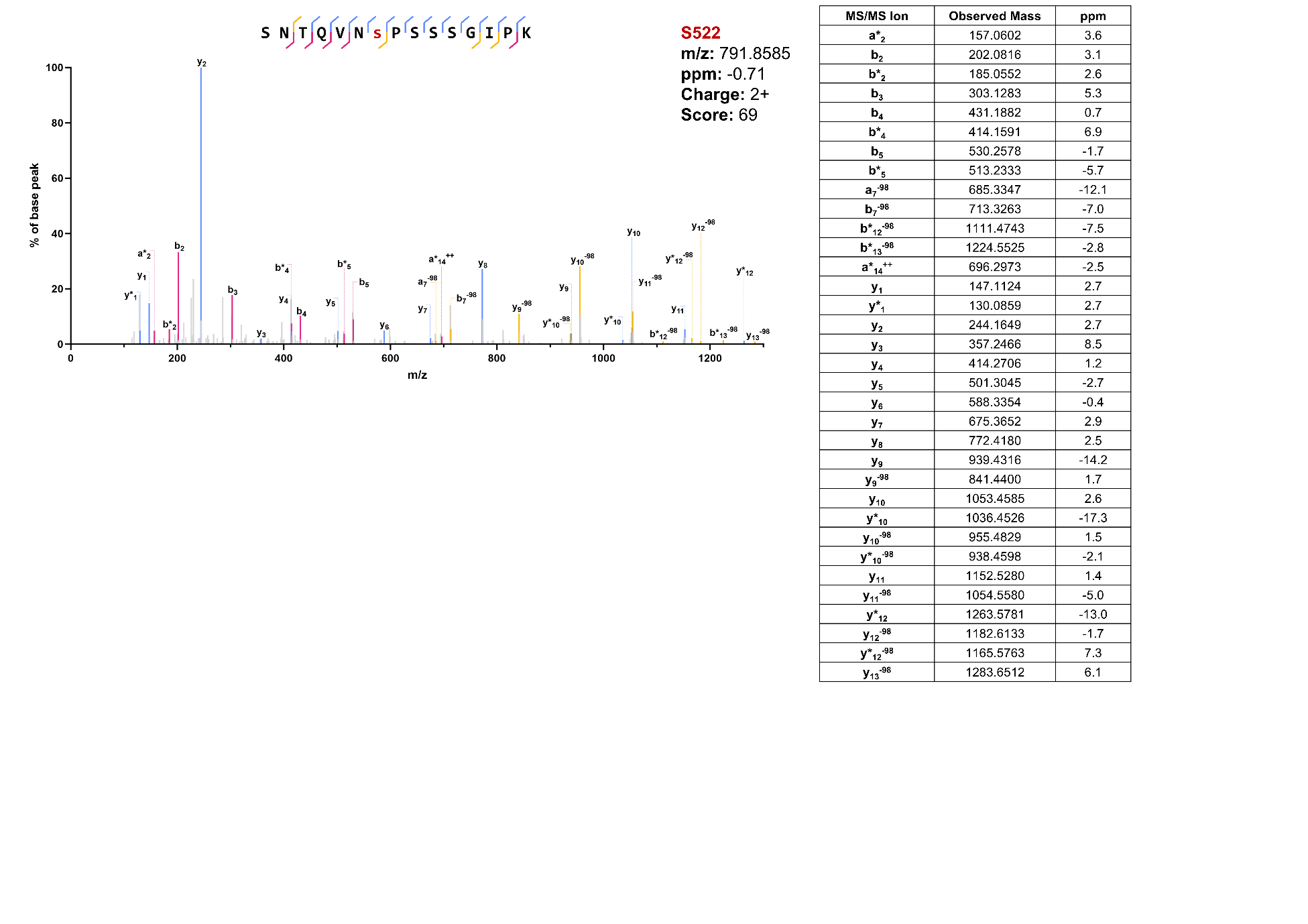


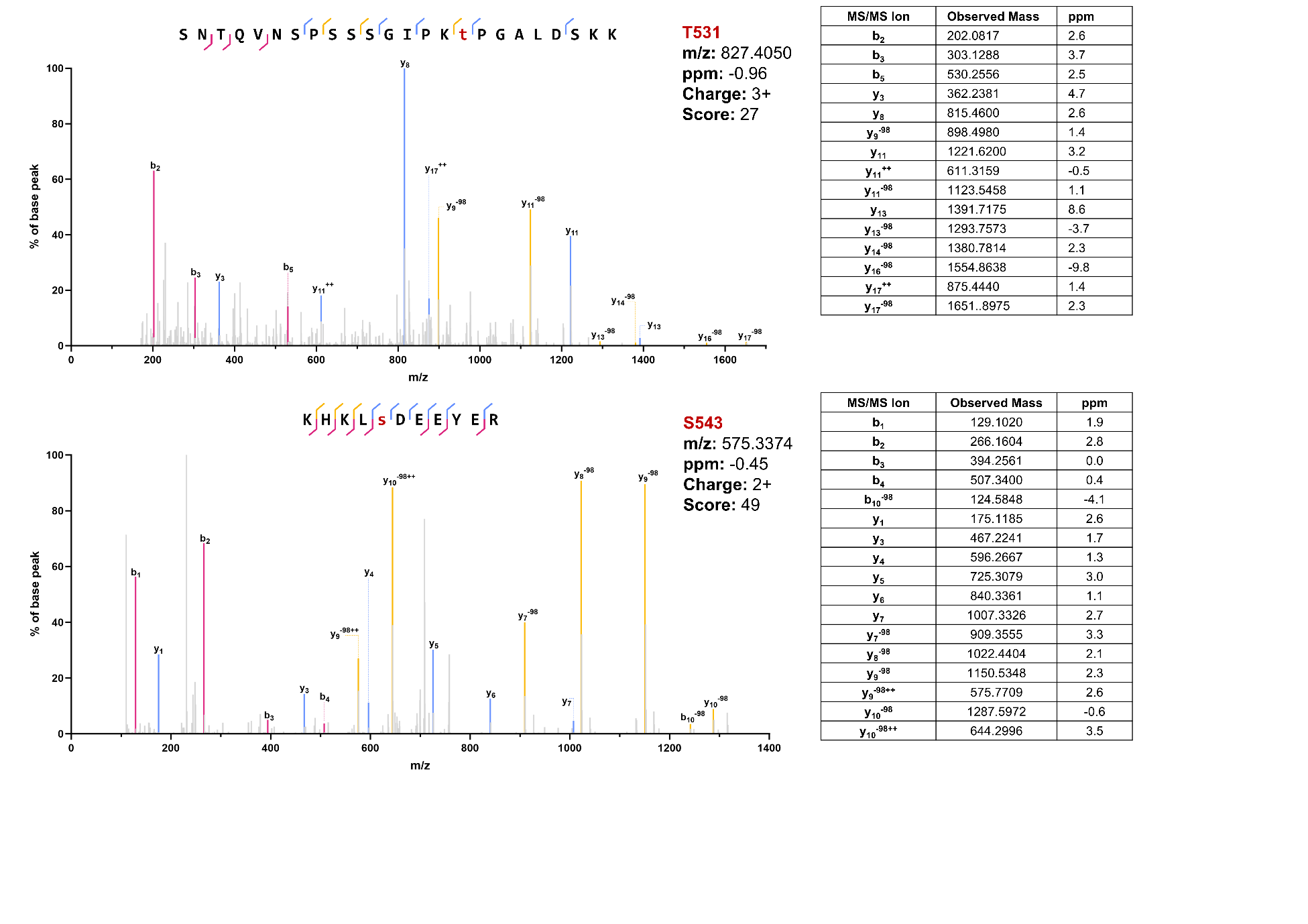


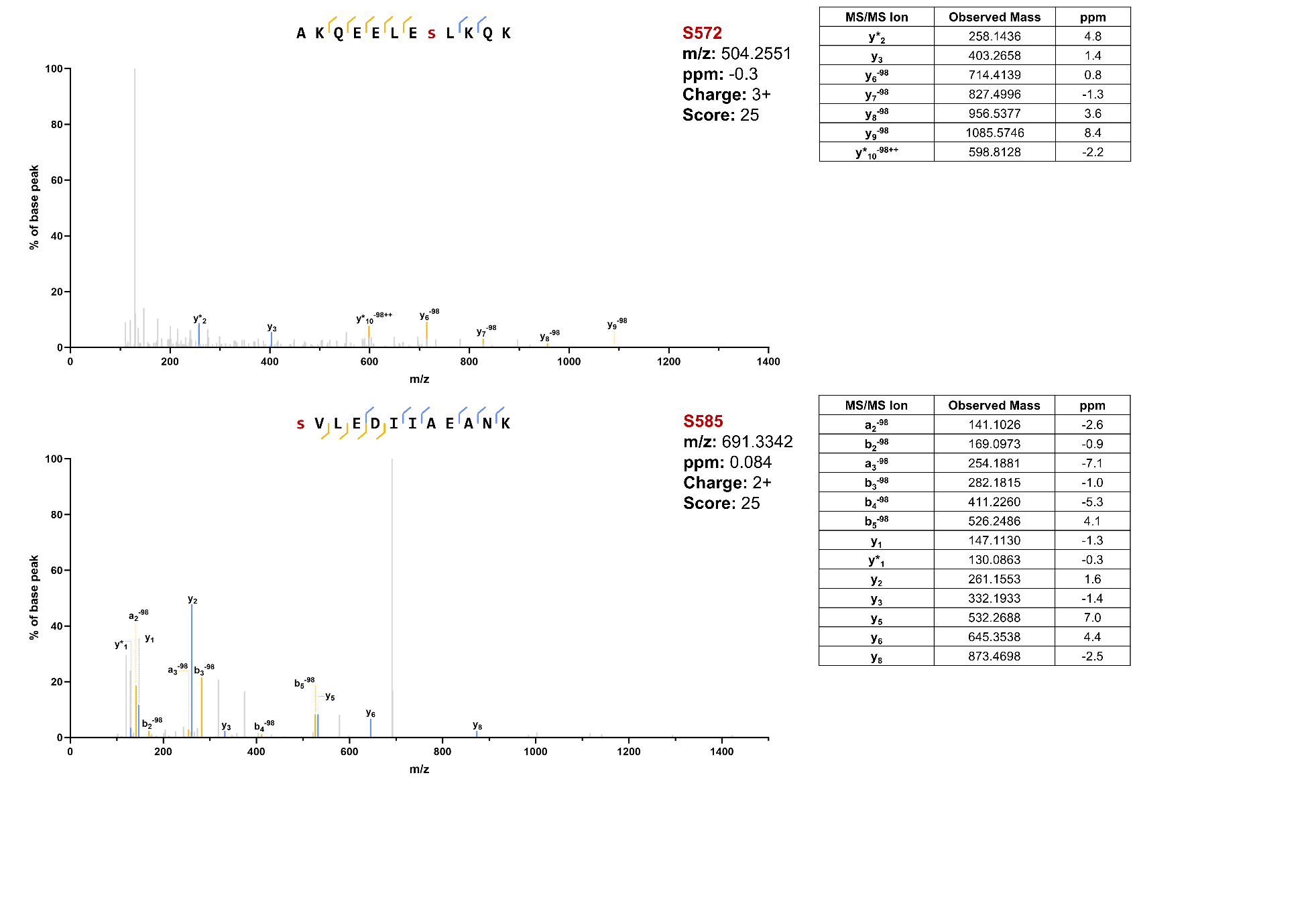


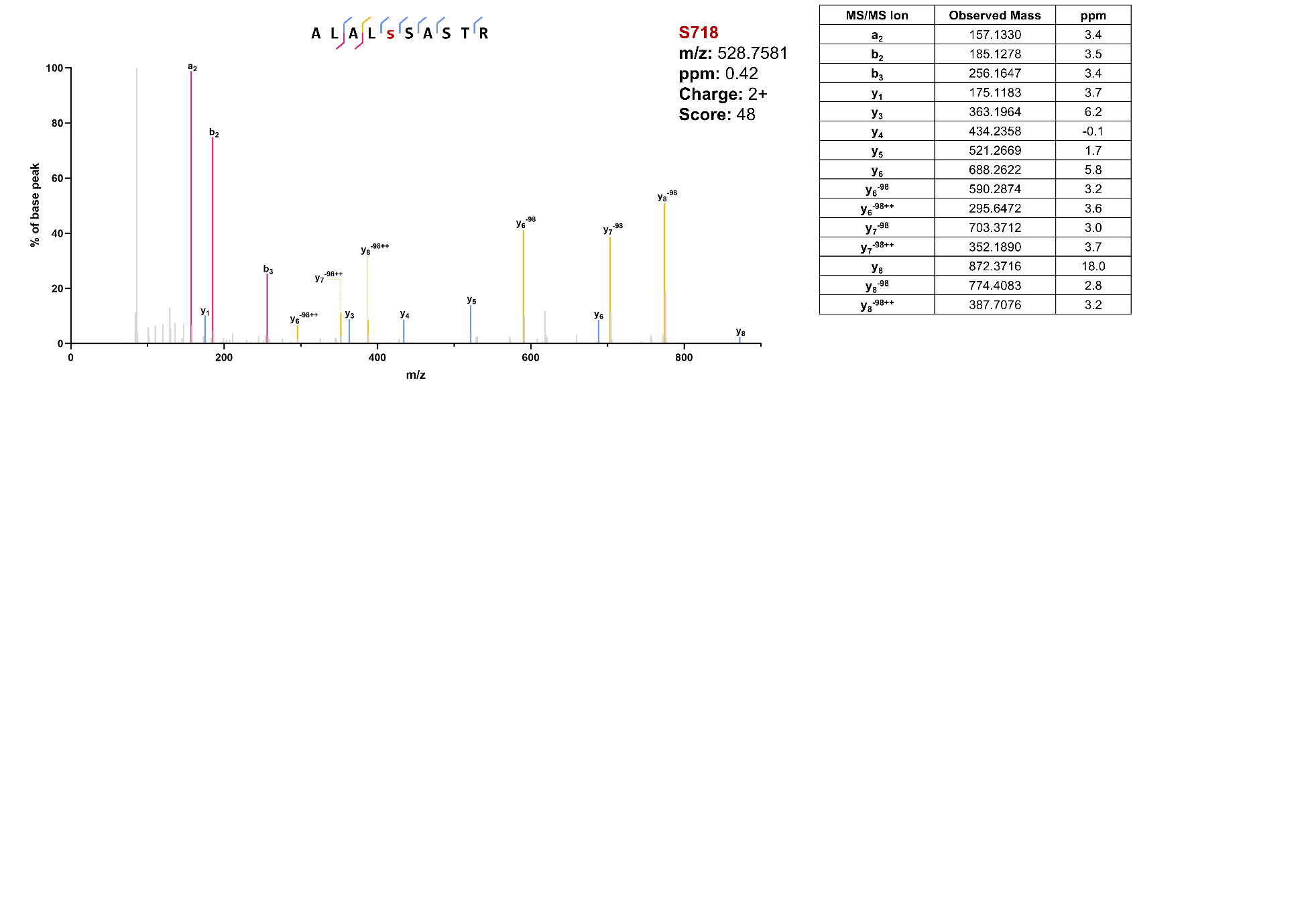


***Figure S2.*** MS2 spectra for localisation of phosphorylation within quantified Set2p phosphopeptides

MS2 spectra used for localisation of phosphosites within quantified Set2p phosphopeptides. b-ions are shown in pink, y-ions are shown in blue and b- and y-ions exhibiting a neutral loss of H_3_PO_4_ (-98) are shown in yellow. The localised phosphosite is shown in the peptide label as lowercase and burgundy. *When there is more than one site indicated, this means the phosphosite could not be localised and any one singly phosphorylated version of the peptide may exist.* MS2 spectra are replicated from the Mascot MS/MS Ions Search output, with only fragment ions used for scoring shown on each spectrum.


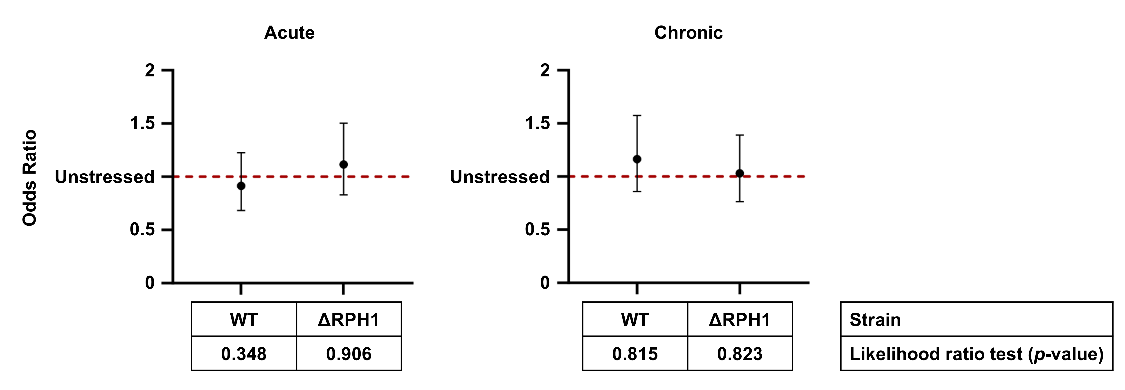


***Figure S3.*** Ordinal logistic regression model of gross methylation levels at H3K36 of the wildtype and RPH1Δ strain in acute salt stress and chronic salt stress compared to no stress

*For each strain, the odds ratio of H3K36 being more methylated in the stress condition compared to the no stress condition is shown.* Experiments were performed on strains in acute 1M NaCl stress (left) and chronic 1M NaCl stress (right). *The odds ratios are presented relative to the no stress condition normalised to 1, which is indicated by a burgundy dotted line. The mean odds ratio of three biological replicates is shown, with error bars indicating the 95% confidence interval. The regression assumes that the odds of moving between adjacent methylation states (e.g. me0 > me1) is equal. The validity of the proportional odds assumption was assessed with a likelihood ratio test, whereby p-value > 0.5 indicates the proportional odds assumption has been satisfied.*


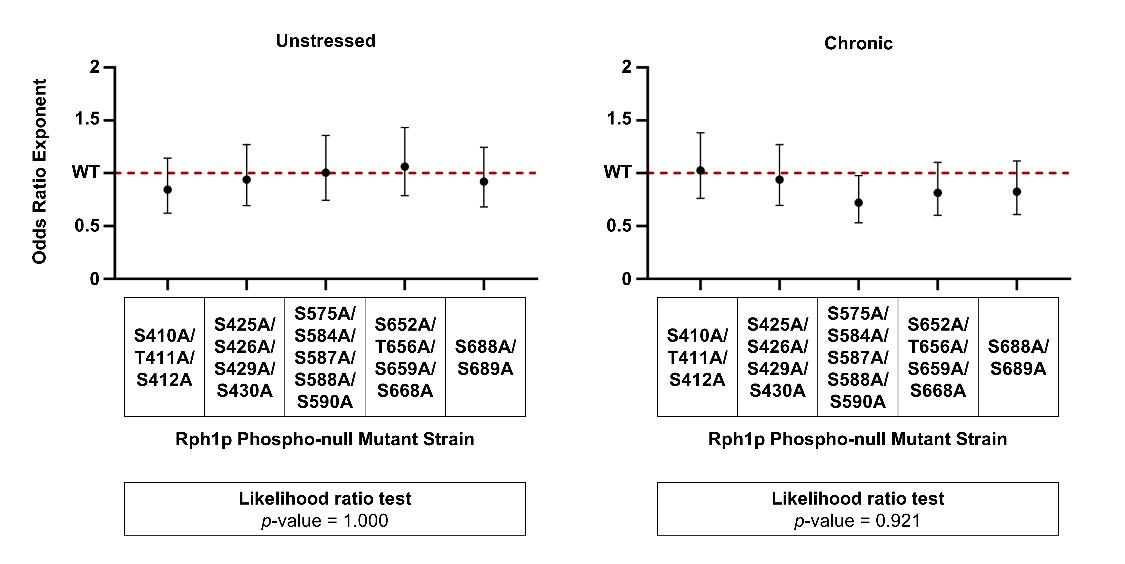


Figure S4. Ordinal logistic regression model of gross methylation levels at H3K36 of the Rph1p phosphonull mutant strains compared to the wildtype strain in no stress and in chronic salt stress

For each condition, the odds ratio of H3K36 being more methylated in the Rph1p phosphonull strain compared to the wildtype strain is shown. The odds ratios are presented relative to the wildtype normalised to 1, which is indicated by a burgundy dotted line. Experiments were performed on strains in the no stress condition (left) and in chronic 1M NaCl stress (right). The mean odds ratio of three biological replicates is shown, with error bars indicating the 95% confidence interval. The regression assumes that the odds of moving between adjacent methylation states (e.g. me0 > me1) is equal. The validity of the proportional odds assumption was assessed with a likelihood ratio test, whereby p-value > 0.5 indicates the proportional odds assumption has been satisfied.


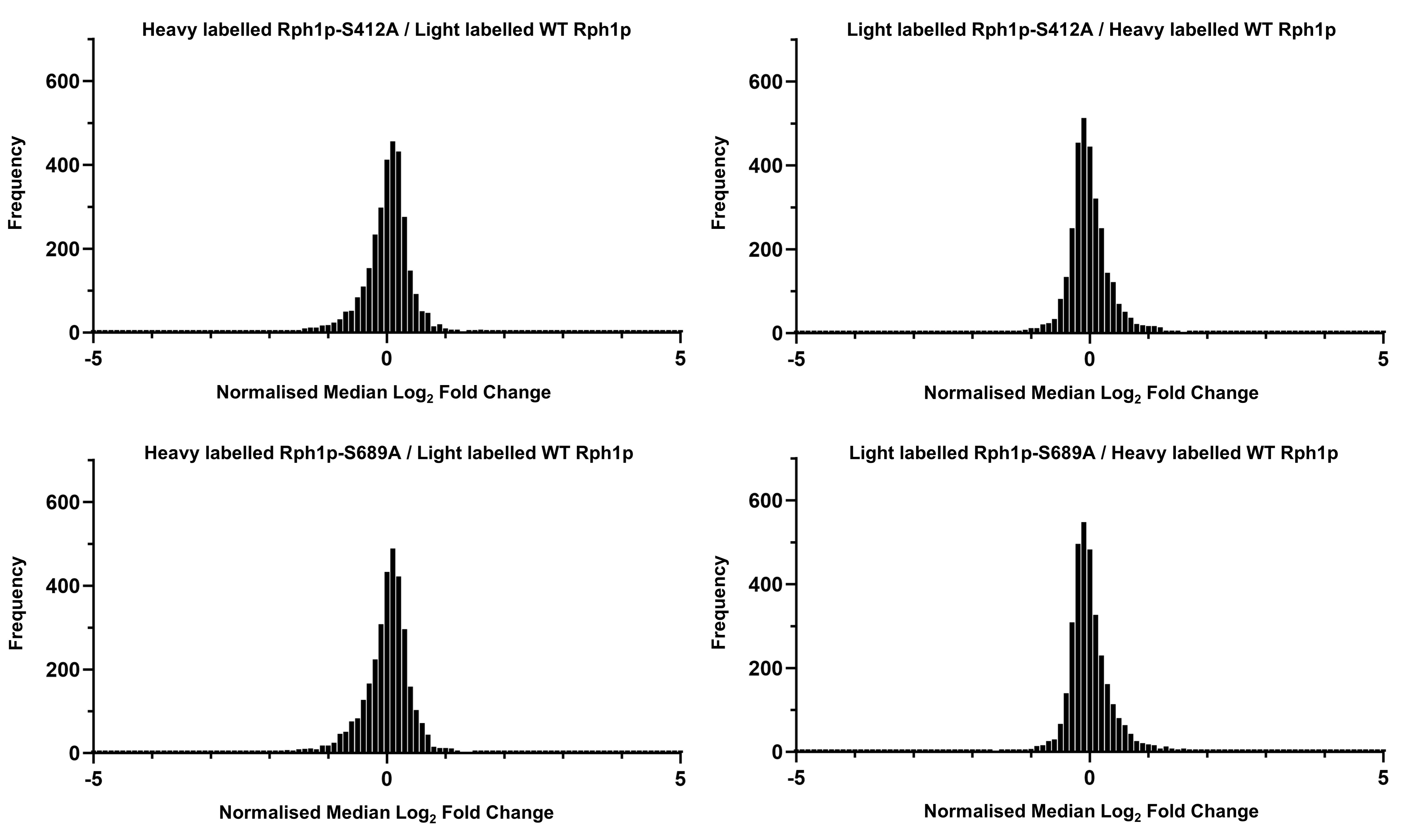


***Figure S5. Distribution of ratios for the 2nSILAC Rph1p-S412A and Rph1p-S689A experiments***

Frequency histograms of the normalised median Log2 transformed heavy-to-light (H/L) ratios for quantified proteins in each label swap replicate for the Rph1p-S412A and Rph1p-S689A experiments. Mass spectrometry data was analysed using the MSFragger SILAC3 workflow to obtain median Log2 H/L ratios. Median Log2 H/L ratios for the reverse labelled replicate (wildtype / mutant) were multiplied by -1 to match the forward label replicate (mutant / wildtype) and both replicates were normalised. *Proteins identified by less than < 2 peptides and contaminant/decoy proteins were removed. Proteins for which ratios were obtained in both biological replicates are shown for each experiment.*


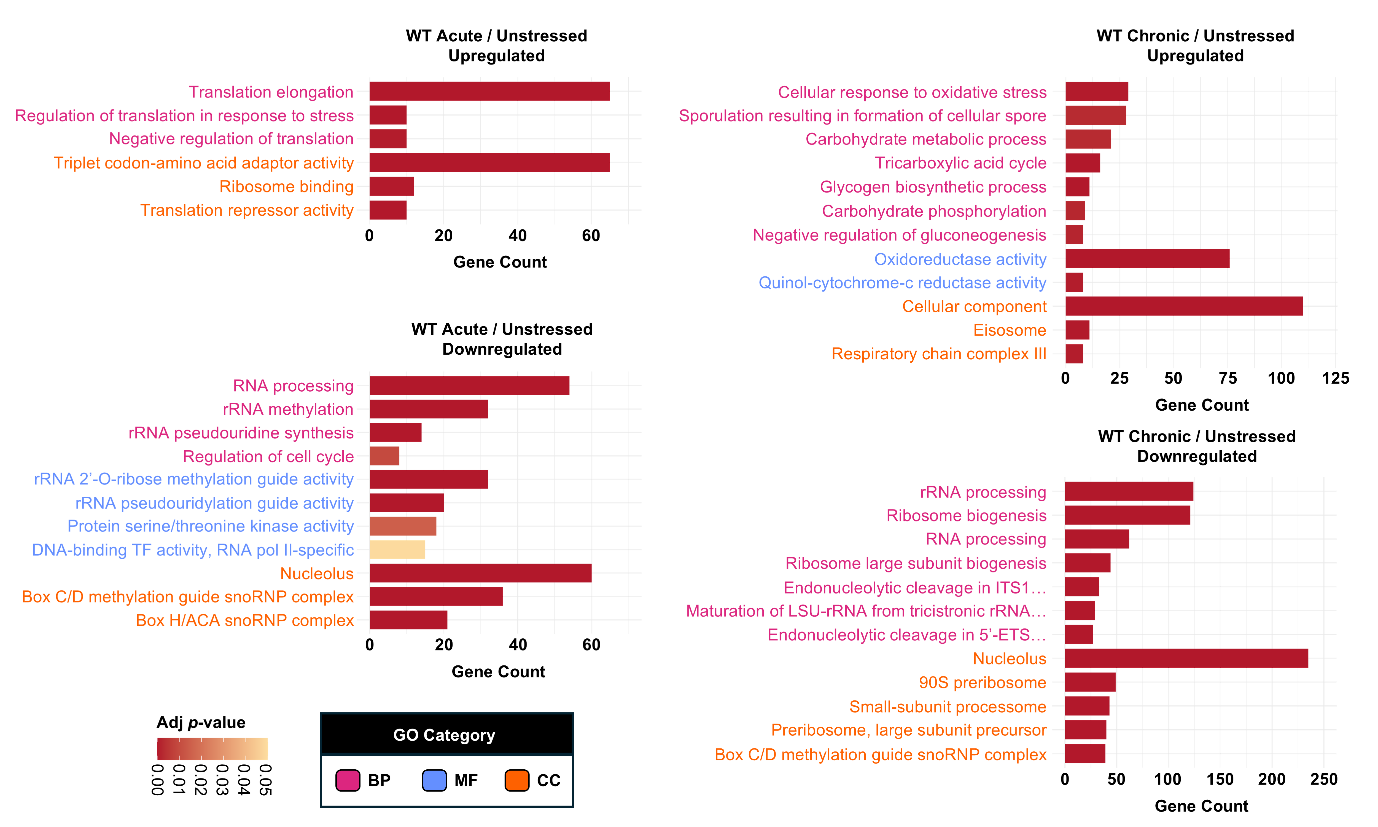
Figure S6. Functional enrichment analysis of differentially expressed genes in the wildtype in acute and chronic salt stress compared to no stress

*Significant shared GO terms of mRNA transcripts upregulated and downregulated in the wildtype in acute 1 M NaCl stress and chronic 1 M NaCl stress compared to no stress; BP = biological process, MF = molecular function, CC = cellular component. The top 12 enriched GO terms by adjusted p-value are presented independent of GO category. Functional enrichment analysis was performed using Enricher with quantified mRNA transcripts set as the background. Statistical analysis was performed using a hypergeometric test with Benjamini-Hochberg FDR correction (adjusted p-value < 0.05).*


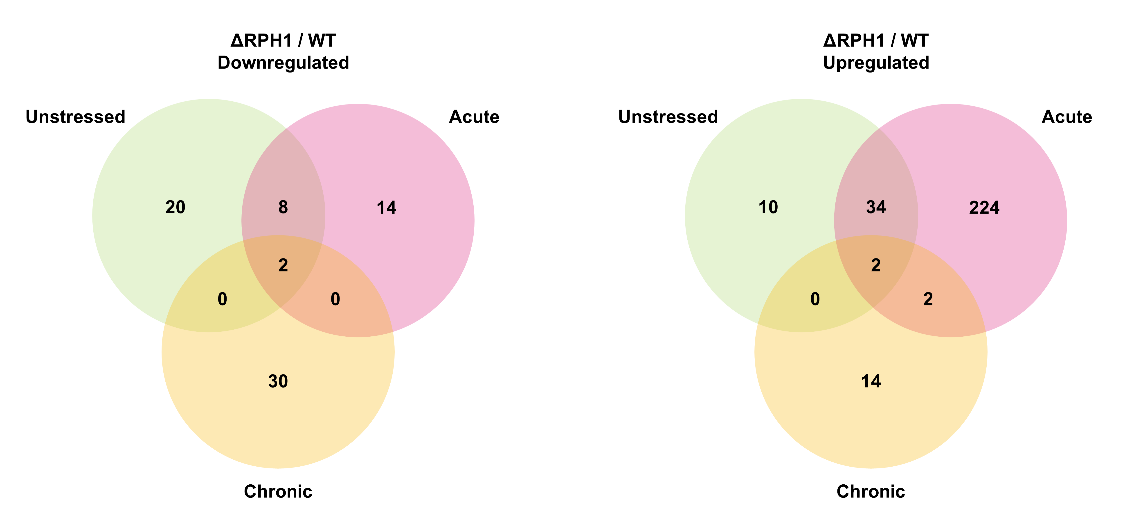


Figure S7. Venn diagrams of downregulated and upregulated genes in the ΔRPH1 compared to wildtype in no stress, acute salt stress and chronic salt stress

Table S1. Name, sequence and purpose of oligonucleotides used in this study

| **Name** | | **Sequence (5′-3′)** | **Purpose** |
| --- | --- | --- | --- |
| RPH1 KO | F | gaaaaaaagaataagactgtcttggtgaggatattcagttgcgtgaaatcA TGCGTACGCTGCAGGTCGAC | Amplification of natNT2 resistance cassette with genomic overhangs for homologous recombination into the RPH1 coding sequence of BY4741 to generate BY4741ΔRPH1 |
|  | R | ttaagagccttcaaaatgagagatctcggtaaacaactggcaatcgtgagTCAATCGATGAATTCGAGCTCG |  |
| pRS426  Back bone | F | GAGAGTGCACCATACCACCAAT | Amplification of the pRS426 back bone |
|  | R | CTGCTCTGATGCCGCATAGTTAAG |  |
| RPH1-6H | F | CTATGCGGCATCAGAGCAGATGACGAAACTAATCGCTC | Amplification of RPH1 from B4741 with a C-terminal 6x histidine tag for cloning into the pRS426 backbone to generate pRS416-RPH1-6H |
|  | R | ggtggtatggtgcactctctcagtgatggtgatggtgatgGTTTAAAGGTGTACTCTGTTG |  |
| pRS426  Check | F | CTTGTCTGTAAGCGGATGC | Confirm incorporation of a gene into pRS426 |
|  | R | CACCGCATAGGGTAATAACTG |  |
| RPH1  S410A | F | AGCAGCTCGCTTAGAGCTACAAGCCCAGATGTGGGTCATTTTTC | Site directed mutagenesis of pRS426-RPH1-6H to generate a S410A substitution |
|  | R | GAAAAATGACCCACATCTGGGCTTGTAGCTCTAAGCGAGCTGCT |  |
| RPH1  T411A | F | AGCAGCTCGCTTAGATCTGCAAGCCCAGATGTGGGTCATTTTTC | Site directed mutagenesis of pRS426-RPH1-6H to generate a T411A substitution |
|  | R | GAAAAATGACCCACATCTGGGCTTGCA GATCTAAGCGAGCTGCT |  |
| RPH1  S412A | F | AGCAGCTCGCTTAGATCTACAGCCCCAGATGTGGGTCATTTTTC | Site directed mutagenesis of pRS426-RPH1-6H to generate a S412A substitution |
|  | R | GAAAAATGACCCACATCTGGGGCTGTAGATCTAAGCGAGCTGCT |  |
| RPH1  S410A/T411A/S412A | F | AGCAGCTCGCTTAGAGCTGCAGCCCCAGATGTGGGTCATTTTTC | Site directed mutagenesis of pRS426-RPH1-6H to generate a tandem S410A/T411A/412A substitution |
|  | R | GAAAAATGACCCACATCTGGGGCTGCAGCTCTAAGCGAGCTGCT |  |
| RPH1  425A/S426A/S429A/S430A | F | CAAACTTTAAATCCAAGGCCGCTGGTGTTGCTGCTCCCTTATTATCACGTATG | Site directed mutagenesis of pRS426-RPH1-6H to generate a tandem S425A/S426A/S429A/S430A substitution |
|  | R | CATACGTGATAATAAGGGAGCAGCAACACCAGCGGCCTTGGATTTAAAGTTTG |  |
| RPH1  S575A/S584A/S587A/S5 88A/S590A | F | ACGAGTTAAGGCTCCCAACATTGTAACATTAAATAT AGCCAGGGAAGCGGCAAGAGCCCCTATAGCTCTG | Site directed mutagenesis of pRS426-RPH1-6H to generate a tandem S575A/S584A/S587A/S588A/S590A substitution |
|  | R | CAGAGCTATAGGGGCTCTTGCCGCTTCCCTGGCTATATTTAATGTTACAATGTTGGGAGCCTTAACTCGT |  |
| RPH1  S652A | F | AGAATACTCAAAAAGGAAGCTCCTGTCGAG ACATCAAAATCA | Site directed mutagenesis of pRS426-RPH1-6H to generate a S652A substitution |
|  | R | TGATTTTGATGTCTCGACAGGAGCTTCCTTTTTGAGTATTCT |  |
| RPH1  S652A/T656A/S659A/S668A | F | CTCAAAAAGGAAGCTCCTGTCGAGGCATCAAAAGCAAATCTTATACTTTCAAAGGTTGCAGCCACTCGCCAAG | Site directed mutagenesis of pRS426-RPH1-6H to generate a tandem S652A/T656A/S659A/S668A substitution |
|  | R | CTTGGCGAGTGGCTGCAACCTTTGAAAGTATAAGATTTGCTTTTGATGCCTCGACAGGAGCTTCCTTTTTGAG |  |
| RPH1  S688A | F | GGACAAAGAACAAGGCGCTTCTCCATTAAACTCTAAG | Site directed mutagenesis of pRS426-RPH1-6H to generate a S688A substitution |
|  | R | CTTAGAGTTTAATGGAGAAGCGCCTTGTTCTTTGTCC |  |
| RPH1  S689A | F | GGACAAAGAACAAGGCTCTGCTCCATTAAACTCTAAG | Site directed mutagenesis of pRS426-RPH1-6H to generate a S689A substitution |
|  | R | CTTAGAGTTTAATGGAGCAGAGCCTTGTTCTTTGTCC |  |
| RPH1  S688A/S689A | F | GGACAAAGAACAAGGCGCTGCTCCATTAAACTCTAAG | Site directed mutagenesis of pRS426-RPH1-6H to generate a tandem S688A/S689A substitution |
|  | R | CTTAGAGTTTAATGGAGCAGCGCCTTGTTCTTTGTCC |  |
| RPH1  Genomic | F | gaaaaaaagaataagactgtcttggtgaggatattcagttgcgtgaa  atcATGACGAAACTAATCGCTCCTTC | Amplification of C-terminal 6x histidine tagged RPH1 and URA3 auxotrophic selection marker with genomic overhangs from mutated pRS426-RPH1-6H vectors for homologous recombination into the RPH1 coding sequence of BY4741 to generate genomically mutated RPH1 strains |
|  | R | ttaagagccttcaaaatgagagatctcggtaaacaactggcaatcgt  gagTTAGTTTTGCTGGCCGCA |  |
| RPH1_A | | GCGATAATTTTAAGAGAATTGCAGA | Colony PCR and confirmation sequencing of genomically mutated RPH1 strains |
| RPH1_Start_Fwd | | GGGGGACTTAACCTTTCTATTAG |  |
| RPH1_412Fwd | | GCAGAATCCGTCAACTTTGC |  |
| RPH1_575Rev | | TGACACGGTTGATGGTGTGG |  |
| URA3_seq_rev_1 | | TCCTTCTGTTCGGAGATTACC |  |
| URA3_Start_Fwd | | CATATAAGGAACGTGCTGCTAC |  |
| RPH1_D | | TTAGTAATCTCGAAGTCTGTGTCCC |  |

Table S2. Name and characteristics of plasmids used in this study

| **Name** | **Description/Features** |
| --- | --- |
| pFA6a-natNT2 | Yeast episomal vector for gene deletion containing the natNT2 selection cassette and Amp^R^ |
| pRS426-RPH1-6H | Yeast episomal shuttling vector containing C-terminal hexahistidine tagged RPH1, URA3 selection cassette and Amp^R^ |
| pRS426-RPH1(S410A)-6H | Yeast episomal shuttling vector containing C-terminal hexahistidine tagged RPH1 with S410A substitution, URA3 selection cassette and Amp^R^ |
| pRS426-RPH1(T411A)-6H | Yeast episomal shuttling vector containing C-terminal hexahistidine tagged RPH1 with T411A substitution, URA3 selection cassette and Amp^R^ |
| pRS426-RPH1(S412A)-6H | Yeast episomal shuttling vector containing C-terminal hexahistidine tagged RPH1 with S412A substitution, URA3 selection cassette and Amp^R^ |
| pRS426-RPH1(S410A/T411A/412A)-6H | Yeast episomal shuttling vector containing C-terminal hexahistidine tagged RPH1 with S410A/T411A/S412A substitutions, URA3 selection cassette and Amp^R^ |
| pRS426-RPH1(S425A/S426A/S429A/S430A)-6H | Yeast episomal shuttling vector containing C-terminal hexahistidine tagged RPH1 with S425A/S426A/S429A/S430A substitutions, URA3 selection cassette and Amp^R^ |
| pRS426-RPH1(S575A/S584A/S587A/S5 88A/S590A)-6H | Yeast episomal shuttling vector containing C-terminal hexahistidine tagged RPH1 with S575A/S584A/S587A/S5 88A/S590A substitutions, URA3 selection cassette and Amp^R^ |
| pRS426-RPH1(S652A)-6H | Yeast episomal shuttling vector containing C-terminal hexahistidine tagged RPH1 with S652A substitution, URA3 selection cassette and Amp^R^ |
| pRS426-RPH1(S652A/T656A/S659A/S668A)-6H | Yeast episomal shuttling vector containing C-terminal hexahistidine tagged RPH1 with S652A/T656A/S659A/S668A substitutions, URA3 selection cassette and Amp^R^ |
| pRS426-RPH1(S688A)-6H | Yeast episomal shuttling vector containing C-terminal hexahistidine tagged RPH1 with S688A substitution, URA3 selection cassette and Amp^R^ |
| pRS426-RPH1(S689A)-6H | Yeast episomal shuttling vector containing C-terminal hexahistidine tagged RPH1 with S689A substitution, URA3 selection cassette and Amp^R^ |
| pRS426-RPH1(S688A/S689A)-6H | Yeast episomal shuttling vector containing C-terminal hexahistidine tagged RPH1 with S688A/S689A substitutions, URA3 selection cassette and Amp^R^ |
| pD1204-RPH1-6H | Yeast overexpression vector containing C-terminal hexahistidine tagged RPH1, GAL1 promoter, URA3 selection cassette and Amp^R^ |
| pD1204-SET2-6H | Yeast overexpression vector containing C-terminal hexahistidine tagged SET2, GAL1 promoter, URA3 selection cassette and Amp^R^ |

***Table S3. Quantified Rph1p phosphopeptides***

| **Phosphosite/s** | **Peptide** | **Charge** |
| --- | --- | --- |
| S72 | K.IK**S**PIQQHISGNK.G | 3+ |
| S412 | R.ST**S**PDVGHFSNFK.S | 3+ |
| S426 | K.SKS**S**GVSSPLLSR.M | 2+ |
| S430 | K.SKSSGVS**S**PLLSR.M | 2+ |
| S459 | R.IS**S**FQEQPLNK.L | 2+ |
| S575  T580  S584 | R.VK**S**PNIV**T**LNI**S**R.E | 2+ |
| S575 | K.**S**PNIVTLNISR.E | 2+ |
| S584 | K.SPNIVTLNI**S**R.E | 2+ |
| S587  S588  S590 | R.E**SS**R**S**PIALNYEAR.Q* | 3+ |
| S590 | R.**S**PIALNYEAR.Q | 2+ |
| S652 | R.ILKKE**S**PVETSK.S  K.KE**S**PVETSK.S | 3+  2+ |
| S668  T669 | K.VA**ST**RQEDSFTSR.N | 3+ |
| S689 | R.NDDLDKEQGS**S**PLNSK.F | 2+ |

*Ambiguous localisation of the phosphosite

**Table S4. Quantified Set2p phosphopeptides**

| **Phosphosite/s** | **Peptide** | **Charge** |
| --- | --- | --- |
| S8  S10 | K.NQSV**S**A**S**EDEK.E* | 2+ |
| T518  S522 | R.SN**T**QVN**S**PSSSGIPK.T | 2+ |
| T531 | R.SNTQVNSPSSSGIPK**T**PGALDSK.K | 3+ |
| S543 | K.KHKL**S**DEEYER.K | 2+ |
| S572 | R.AKQEELE**S**LKQK.L | 3+ |
| S585 | K.**S**VLEDIIAEANK.Q | 2+ |
| S718  S719  S721 | K.ALAL**SS**A**S**TR.M | 2+ |

*Ambiguous localisation of the phosphosite

Table S5. Efficiency of incorporation of heavy amino acid isotopes (arginine, lysine and proline) into yeast lysate based on peptide spectral matches

*Mass spectra were searched in MSFragger SILAC3 workflow using IonQuant for MS1 quantification with heavy labelling Arg10 and Lys8, and light labelling Arg0 and Lys10 selected, along with match between runs. Modifications included carbamidomethyl (C) (fixed), oxidation (M) (variable), acetylation (N-term) (variable), Arg10 (R) (variable), Lys8 (K) (variable) and Pro6 (P) (variable).*

|  | **Arg10/Lys6**  **PSMs** | **Arg0/Lys0**  **PSMs** | **Pro6**  **PSMs** | **Pro0**  **PSMs** |
| --- | --- | --- | --- | --- |
| **WT Rph1p** | 12783 (99.13%) | 112 | 825 (6.40%) | 12070 |
| **Rph1p-S412A** | 13685 (99.20%) | 111 | 885 (6.41%) | 12911 |
| **Rph1p-S689A** | 11959 (99.18%) | 99 | 721 (5.98%) | 11337 |

Table S8. Significantly (adjusted p-value < 0.05) downregulated (mean Log2 fold change ≤ 0.8) transcripts in the Rph1p-S689A strain compared to wildtype strain grown in chronic 1 M NaCl

| **Systematic Name** | **Standard Name** | **Mean Log2 Fold Change** | **Description^a^** |
| --- | --- | --- | --- |
| YDR504C | SPG3 | -0.87248 | Protein required for survival in high temperature during stationary phase |
| YDR467C | - | -1.12359 | Dubious open reading frame unlikely to encode a functional protein |
| YNCD0010C | SNR47 | -1.06098 | C/D box snoRNA that guides 2'-O-methylation of LSU rRNA at A2220 and SSU rRNA at A619 |
| YDR220C | - | -1.84328 | Dubious open reading frame unlikely to encode a functional protein |
| YDR261C-D | - | -1.11535 | Retrotransposon TYA Gag and TYB Pol genes |
| YOR102W | - | -1.51464 | Dubious open reading frame unlikely to encode a functional protein |
| YOR105W | - | -1.35480 | Protein of unknown function |
| YOR302W | - | -1.14052 | Arg attenuator peptide that regulates translation of the CPA1 mRNA |
| YOR192C-C | - | -1.30643 | Protein of unknown function |
| YOL103W-B | - | -1.41952 | Retrotransposon TYA Gag and TYB Pol genes |
| YGR240C-A | - | -0.88841 | Putative protein of unknown function |
| YGR025W | - | -2.35007 | Putative protein of unknown function |
| YNCG0001C | RME3 | -1.09760 | ncRNA antisense transcript that represses the meiosis-specific ZIP2 gene and is expressed in haploid cells under sporulation inducing conditions |
| YGR122C-A | - | -1.08437 | Dubious open reading frame unlikely to encode a functional protein |
| YGL226C-A | OST5 | -0.93427 | ζ-subunit of the oligosaccharyltransferase complex of the ER lumen which catalyses Asn-linked glycosylation of newly synthesized proteins |
| YGR039W | - | -0.87572 | Putative protein of unknown function |
| YNCG0014C | SNR39 | -1.19311 | C/D box snoRNA that guides 2'-O-methylation of LSU rRNA at A807 |
| YNCG0015C | SNR39B | -0.96743 | C/D box small snoRNA that guides 2'-O-methylation of LSU rRNA at G805 |
| YGR174W-A | - | -2.08261 | Putative protein of unknown function |
| YNCL0041C | SNR61 | -1.97174 | C/D box small snoRNA that guides 2'-O-methylation of LSU rRNA at A1133 |
| YLR311C | - | -1.17982 | Dubious open reading frame unlikely to encode a functional protein |
| YNCL0043C | SNR57 | -1.55767 | C/D box snoRNA that guides 2'-O-methylation of SSU rRNA at G1572 |
| YLR456W | - | -0.90283 | Protein of unknown function |
| YNCP0013C | SNR51 | -0.99767 | C/D box snoRNA that guides 2'-O-methylation of LSU rRNA at U2729 and SSU rRNA at A100 |
| YPL119C-A | - | -1.50929 | Protein of unknown function |
| YNCP0003W | SNR59 | -1.06121 | C/D box snoRNA that guides 2'-O-methylation of LSU rRNA at A807 |
| YNCM0031W | SNR11 | -1.08992 | H/ACA box snoRNA that guides pseudouridylation of LSU rRNA at U2416 |
| YNCM0012W | SNR77 | -1.60721 | C/D box snoRNA that guides 2'-O-methylation of SSU rRNA at U578 |
| YNCM0026C | SNR24 | -0.96447 | C/D box snoRNA that guides 2'-O-methylation of LSU rRNA at C1437, C1449 and C1450 |
| YMR001C-A | - | -2.01065 | Putative protein of unknown function |
| YMR323W | ERR3 | -0.84372 | Enolase that catalyses the conversion of 2-phosphoglycerate to phosphoenolpyruvate |
| YNCM0005C | SNR54 | -1.56955 | C/D box snoRNA that guides 2'-O-methylation of SSU rRNA at position A974 |
| YBL048W | RRT1 | -0.97192 | Putative protein of unknown function |
| YBR292C | - | -1.06975 | Putative protein of unknown function |
| YNR075W | COS10 | -0.92411 | Endosomal protein involved in turnover of plasma membrane proteins |
| YNL146W | - | -1.13063 | Putative protein of unknown function |
| YNR025C | - | -0.83760 | Dubious open reading frame unlikely to encode a functional protein |
| YNCN0014W | SNR66 | -1.21156 | C/D box snoRNA that guides 2'-O-methylation of LSU rRNA at U2417 |
| YNCN0018W | SNR49 | -1.65049 | H/ACA box snoRNA that guides pseudouridylation of LSU rRNA at U990 and SSU rRNA at U120, U211 and U302 |
| YNL109W | - | -0.93976 | Dubious open reading frame unlikely to encode a functional protein |
| YNCJ0007C | SNR37 | -1.17435 | H/ACA box snoRNA that guides pseudouridylation of LSU rRNA at U2944; involved in ribosome synthesis and has a structural role in early pre-60S pathway |
| YNCJ0004C | SNR190 | -0.95491 | C/D box snoRNA predicted to guide 2'-O-methylation of LSU rRNA at G2395 |
| YKR004C | ECM9 | -0.96133 | α-subunit of geranylgeranyltransferase type III; required with β-subunit Bet2p to transfer a geranylgeranyl group to mono-farnsylated Ykt6p, regulating membrane localisation and its function in cell wall integrity and autophagy; involved in cell wall maintenance and functions in a regulatory pathway determining cell division readiness under stress |
| YKL115C | - | -1.44248 | Dubious open reading frame unlikely to encode a functional protein |
| YNCK0009W | SNR38 | -1.38198 | C/D box snoRNA that guides 2'-O-methylation of LSU rRNA at G2815 |
| YKL136W | - | -1.33387 | Dubious open reading frame unlikely to encode a functional protein |
| YER138W-A | - | -1.68695 | Putative protein of unknown function |
| YER175W-A | - | -2.18674 | Putative protein of unknown function |
| YNCE0002W | SNR67 | -1.31785 | C/D box snoRNA that guides 2'-O-methylation of LSU rRNA at G2619 and U2724 |
| YHR050W-A | - | -2.14455 | Protein of unknown function |
| YCL054W-A | RDT1 | -0.90529 | Small gene in chromosome III recombination enhancer that is activated during mating-type switching and bound by condensin complex; promoter functions as locus control region that regulates both transcription and long-range chromatin interactions |
| YNCC0011W | SNR65 | -2.08859 | C/D box snoRNA that guides 2'-O-methylation of LSU rRNA at U2347 |
| YCR024C-B | - | -0.87780 | Protein of unknown function |
| YCR018C-A | - | -1.31600 | Putative protein of unknown function |
| YFL019C | - | -1.1852 | Putative protein of unknown function |

^a^ Descriptions derived from (1, 2)

References

1. The UniProt Consortium (2023) UniProt: the universal protein knowledgebase in 2023. *Nucleic Acids Research*. **51**, D523–D531

2. Engel, S. R., Aleksander, S., Nash, R. S., Wong, E. D., Weng, S., Miyasato, S. R., Sherlock, G., and Cherry, J. M. (2025) Saccharomyces Genome Database: advances in genome annotation, expanded biochemical pathways, and other key enhancements. *Genetics*. **229**, iyae185
